# A drug library screen identifies Carbenoxolone as novel FOXO inhibitor that overcomes FOXO3-mediated chemoprotection in high-stage neuroblastoma

**DOI:** 10.1038/s41388-019-1044-7

**Published:** 2019-10-07

**Authors:** Stefan Salcher, Gilles Spoden, Judith Hagenbuchner, Sebastian Führer, Teresa Kaserer, Martin Tollinger, Petra Huber-Cantonati, Thomas Gruber, Daniela Schuster, Ronald Gust, Heinz Zwierzina, Thomas Müller, Ursula Kiechl-Kohlendorfer, Michael J. Ausserlechner, Petra Obexer

**Affiliations:** 1 Department of Pediatrics II, Medical University Innsbruck, Innsbruck, Austria; 2 Tyrolean Cancer Research Institute, Innsbruck, Austria; 3 Institute of Organic Chemistry, CMBI - Center for Molecular Biosciences Innsbruck, University of Innsbruck, Innsbruck, Austria; 4 Institute of Pharmacy/Pharmaceutical Chemistry, CMBI - Center for Molecular Biosciences Innsbruck, University of Innsbruck, Innsbruck, Austria; 5 Division for Translational Cell Genetics, Medical University Innsbruck, Innsbruck, Austria; 6 Institute of Pharmacy, Department of Pharmaceutical and Medicinal Chemistry, Paracelsus Medical University Salzburg, Salzburg, Austria; 7 Department of Internal Medicine V, Medical University Innsbruck, Innsbruck, Austria; 8 Department of Pediatrics I, Medical University Innsbruck, Innsbruck, Austria

**Keywords:** Cancer, Drug discovery

## Abstract

The transcription factor FOXO3 has been associated in different tumor entities with hallmarks of cancer, including metastasis, tumor angiogenesis, maintenance of tumor-initiating stem cells, and drug resistance. In neuroblastoma (NB), we recently demonstrated that nuclear FOXO3 promotes tumor angiogenesis in vivo and chemoresistance in vitro. Hence, inhibiting the transcriptional activity of FOXO3 is a promising therapeutic strategy. However, as no FOXO3 inhibitor is clinically available to date, we used a medium-throughput fluorescence polarization assay (FPA) screening in a drug-repositioning approach to identify compounds that bind to the FOXO3-DNA-binding-domain (DBD). Carbenoxolone (CBX), a glycyrrhetinic acid derivative, was identified as a potential FOXO3-inhibitory compound that binds to the FOXO3-DBD with a binding affinity of 19 µM. Specific interaction of CBX with the FOXO3-DBD was validated by fluorescence-based electrophoretic mobility shift assay (FAM-EMSA). CBX inhibits the transcriptional activity of FOXO3 target genes, as determined by chromatin immunoprecipitation (ChIP), DEPP-, and BIM promoter reporter assays, and real-time RT-PCR analyses. In high-stage NB cells with functional TP53, FOXO3 triggers the expression of SESN3, which increases chemoprotection and cell survival. Importantly, FOXO3 inhibition by CBX treatment at pharmacologically relevant concentrations efficiently repressed FOXO3-mediated SESN3 expression and clonogenic survival and sensitized high-stage NB cells to chemotherapy in a 2D and 3D culture model. Thus, CBX might be a promising novel candidate for the treatment of therapy-resistant high-stage NB and other “FOXO-resistant” cancers.

## Introduction

Chemotherapy resistance is one of the biggest challenges in the treatment of aggressive neuroblastoma (NB), a childhood tumor that develops from precursor cells of the neural crest during embryogenesis [1]. The transcription factor FOXO3, a member of the forkhead box O (FOXO) superfamily, is involved in a variety of cellular outcomes ranging from apoptosis induction to drug resistance and longevity [2, 3]. FOXO3 has been characterized as tumor suppressor gene based on its anti-proliferative and pro-apoptotic functions [2]. However, an increasing number of studies point out the “dark side” of FOXO3 and describe its potential oncogenic properties in different cancer types, including chronic myeloid leukemia (CML), acute myeloid leukemia (AML), breast cancer, glioblastoma, and pancreatic cancer [4–17]. Our own studies demonstrate that nuclear FOXO3 promotes tumor angiogenesis in vivo [18] and chemoresistance in vitro [19] in aggressive NB.

In neuronal tumor cells the protein-kinase-B (PKB) is frequently hyper-activated causing phosphorylation and functional inactivation of FOXO3 [20, 21]. However, in post-chemotherapy patient biopsies FOXO3 localizes to the nucleus even when phosphorylated at threonine-32, a known PKB site [18]. Cellular stress, e.g., triggered by chemotherapy, overrides growth factor-mediated phosphorylation/inactivation of FOXO3 by PKB, which results in re-activation of FOXO3 and correlates with stage M disease and adverse clinical prognosis [18].

In CML, FOXO3 plays an essential role for long-term maintenance of leukemia-initiating stem cells [10] that are resistant to tyrosine kinase inhibitor (TKI) therapy, preventing complete molecular response, and causing recurrence of CML [22]. TKI-mediated nuclear translocation of FOXO3 leads to cell cycle arrest [23] and further induces drug resistance of CML cells via regulation of the multidrug resistance gene 1 (MDR1) and the phosphatidylinositol-4,5-bisphosphate-3-kinase catalytic subunit alpha (PIK3CA) [4, 5]. Also, in glioma stem cells FOXO3 is involved in radiotherapy resistance, as inhibition of FOXO3 enhances the response to radiotherapy [24]. Sykes et al. observed active FOXO3 in AML patient samples and demonstrated that conditional deletion of FOXOs in an AML mouse model reduces leukemia-initiating cell function in vivo, improves animal survival, and mediates myeloid maturation and AML cell death [12]. Recent studies indicate that FOXO3 triggers chemoresistance in glioblastoma via the regulation of β-catenin [16] and elevated FOXO3 expression is associated with poor patient prognosis [17]. Yu et al. identified FOXO3 as a key regulator in cetuximab resistance in metastatic colorectal cancer [25]. Drug resistance is also mediated by the FOXO-FOXM1 axis thereby promoting tumorigenesis and cancer progression [26]. In breast cancer FOXO3 modulates tumor progression by promoting cell invasion via the induction of matrix metalloproteinases [7] and in pancreatic cancer active FOXO3 correlates with poor prognosis as it acts as an essential regulator of CD44^+^ stem cells [14, 15]. In line, Zhou et al. recently demonstrated that FOXO3 is highly expressed in pancreatic cancer tissues. The downregulation of FOXO3 suppresses invasion and migration, induces G0/G1 phase arrest, and promotes apoptosis of pancreatic carcinoma cells in vitro [27].

The aforementioned studies indicate that inhibiting the transcriptional activity of FOXO3 is a highly promising approach for novel therapeutic interventions in different forms of cancer. Especially eradicating the FOXO3 activity in tumor stem cells would provide markedly improved therapeutic benefits to patients. As currently no FOXO3-inhibitory drug is available for clinical purpose, this study was designed to identify by a drug-repositioning strategy, clinically-approved compounds that bind to the FOXO3-DNA-binding-domain (DBD) and thereby silence its transcriptional activity. By screening the Prestwick Chemical Library^®^ containing 1120 FDA-approved drugs, carbenoxolone disodium salt (CBX) was identified as potential FOXO3-inhibitory compound. CBX silenced the transcriptional activity of FOXO3 at a pharmacologically relevant concentration, efficiently repressed the pro-survival function of FOXO3 by inhibiting the detoxifying protein SESN3, and sensitized high-stage NB cells to chemotherapy. CBX is the first identified FOXO3 inhibitor and represents a promising drug for rapid entry into the clinic for NB treatment, as well as other tumors in which inhibition of FOXO3 could be a potential therapeutic strategy.

## Results

### The small molecular weight compound CBX displaces the IRE-FAM oligonucleotide from the FOXO3-DBD

**Fig. 1:**
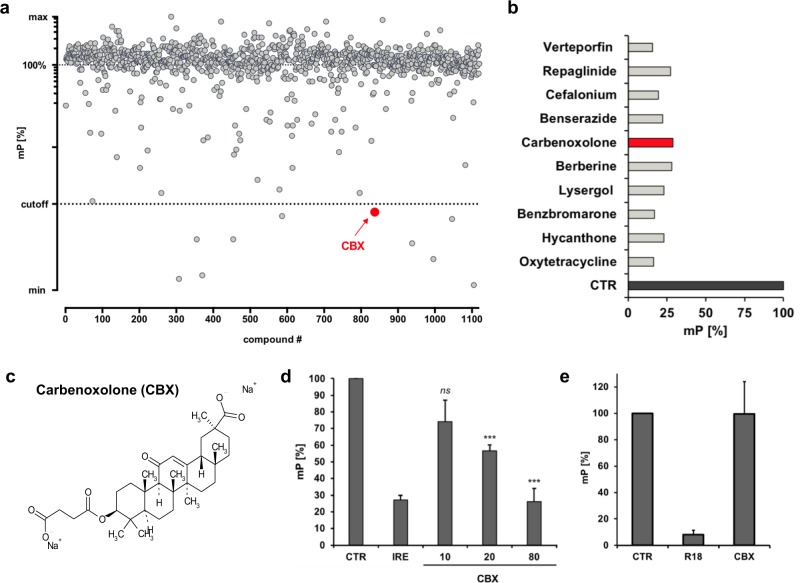
The compound CBX interacts with the recombinant FOXO3-DBD protein. **a** Screening of the 1120 substance Prestwick Chemical Library^®^ for compounds that bind to the FOXO3-DBD by FPA. 100 nM recombinant FOXO3-DBD protein, 5 nM fluorescence-labeled IRE oligonucleotide (IRE-FAM), and 1 µl of each compound were incubated for 30 min at room temperature and analyzed in the chameleon plate reader. **a**, **b** 10 compounds that potentially bind to the FOXO3-DBD protein were identified (cut-off < 31% mP-value). **c** Chemical structure of CBX. **d** Analyses of the dose-dependent interaction of CBX with the FOXO3-DBD protein (20 nM) by FPA using 5 nM IRE-FAM oligonucleotide in combination with 0.5 µM unlabeled IRE oligonucleotide or with indicated concentrations of CBX (µM). Shown are means ± s.e.m. of three independent experiments. Statistical analysis was done using the Student’s unpaired *t* test; ***P < 0.01 compared with the negative control (CTR). **e** Analyses of specific binding of CBX to the FOXO3-DBD by an alternative FPA system that measures the binding of an R18 peptide (10 nM) to recombinant 14–3–3 sigma protein (100 nM). In this setting CBX did not quench the mP-value at a concentration of 163 µM, indicating specific binding of CBX to the FOXO3-DBD protein

The Prestwick Chemical Library^®^ – a collection of 1120 molecules comprising 100% approved drugs (FDA, EMEA) selected for their high chemical and pharmacological diversity – was screened by fluorescence polarization assay (FPA) using the recombinant GST-His-purified FOXO3-DBD (residues 156−269) protein (Supplementary Fig. S1) and a fluorophore-labeled oligonucleotide containing the insulin-responsive element (IRE; Fig. 1a) [28]. The binding of the IRE-FAM oligonucleotide to the FOXO3-DBD protein slows down rotation of the FAM-labeled oligonucleotide in suspension and thus increases polarization upon excitation with polarized light (negative control, CTR). Binding of a compound or of excess of unlabeled IRE oligonucleotide (0.5 µM, positive control, IRE) to the FOXO3-DBD protein causes an increase of unbound, highly rotating IRE-FAM oligonucleotide and depolarisation of the emitted light, thereby leading to a reduction of the millipolarization (mP) value.

Out of the 1120 screened compounds ten showed binding to the FOXO3-DBD at a cut-off <31% mP-value (Fig. 1b). CBX (Fig. 1c), a water-soluble disodium salt of glycyrrhetinic acid hemisuccinate, which is clinically used for the treatment of peptic, esophageal, and oral ulceration [29], showed the most potent effect on FOXO3 inhibition in our screening (Supplementary Fig. S2) and was therefore selected for further studies to investigate its efficacy to inhibit the transcriptional activity of FOXO3 and its potential therapeutic use in NB treatment.

At the used FPA conditions, CBX showed a dose-dependent interaction with the recombinant FOXO3-DBD protein; 20 µM of CBX was sufficient to significantly repress binding of the IRE-FAM oligonucleotide to the FOXO3-DBD protein (Fig. 1d). The most prominent reduction of the mP-value was achieved with 80 µM CBX reaching the same mP-value as with a 100-fold (0.5 µM) higher concentration of unlabeled IRE oligonucleotide (IRE, Fig. 1d). Unspecific inhibition of protein-ligand interaction by CBX was further analyzed by a second FPA system that measures the binding of an R18 peptide to recombinant 14-3-3 sigma protein [30, 31]. In this setting, CBX did not affect fluorescence polarization at a concentration of 163 µM (Fig. 1e), supporting the notion that CBX specifically binds to the FOXO3-DBD protein.

Of note, all FOXO proteins show a high degree of amino-acid sequence identity in their highly conserved forkhead DBD [32]. To determine whether CBX also targets other FOXO family members via interaction with their respective forkhead DBD, FPA were performed. FPA experiments using the GST-His-purified FOXO1-DBD (residues 159–272), FOXO6-DBD (residues 87–200) as well as His-purified FOXO4-DBD (residues 82–207) proteins (Supplementary Fig. S3a), indicated that CBX binds in addition to the FOXO3-DBD, also the FOXO1-DBD, the FOXO4-DBD, as well as the FOXO6-DBD, respectively (Supplementary Fig. S3b). At the applied FPA conditions, CBX interacted with the distinct FOXO-DBD proteins in a dose-dependent manner. The most prominent effect was detected using the FOXO1-DBD protein – 10 µM of CBX were sufficient to quench the mP-value to control level (Supplementary Fig. S3b). However, as demonstrated by immunoblot experiments, exclusively endogenous FOXO3, and neither FOXO1, FOXO4, nor FOXO6, is expressed in the investigated high-stage NB cell lines (Supplementary Fig. S3c). Consequently, the main focus of the present study was to examine the efficacy of CBX to inhibit FOXO3 in order to overcome FOXO3-dependent chemoprotection in NB.

As CBX represents a hemisuccinate derivative of enoxolone (Supplementary Fig. S4a), we also investigated the interaction of enoxolone with the recombinant FOXO3-DBD protein. Importantly, enoxolone did not repress binding of the IRE-FAM oligonucleotide to the FOXO3-DBD protein at a concentration of 80 µM (Supplementary Fig. S4b), indicating that enoxolone does not bind to the FOXO3-DBD.

In concordance, docking studies of CBX and enoxolone into the DNA-interaction site of the FOXO3-DBD (PDB entry 2UZK [33]) retrieved a higher (i.e., better) docking score for CBX compared with enoxolone (Supplementary Fig. S4c). This may be ascribed to an additional ionic interaction formed between the hemisuccinate carboxylate of CBX and Arg249. This residue is also involved in ionic interactions with the phosphate backbone upon binding of DNA, and CBX with its additional negatively charged carboxylate group may therefore be better suited to mimic the interactions between the FOXO3-DBD and its native ligand.

### Biochemical characterization of CBX

**Fig. 2:**
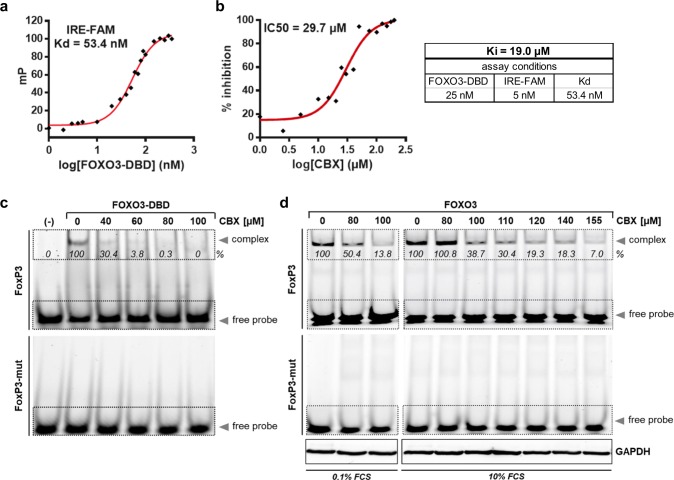
Biochemical characterization of the interaction between CBX and the FOXO3-DBD protein. **a** The dissociation constant (K_d_) for the protein/ligand pair FOXO3-DBD/IRE-FAM was determined by FPA using the IRE-FAM oligonucleotide (5 nM) and increasing concentrations of the FOXO3-DBD protein (1–350 nM). **b** The IC50-value of CBX was determined by FPA in a competitive binding experiment with constant concentrations of the FOXO3-DBD protein (25 nM) and the IRE-FAM oligonucleotide (5 nM). The IC50-value was calculated by nonlinear least-square analysis. The binding affinity (K_i_) value of CBX was calculated by the equation of Nikolovska–Coleska. **c** Binding of CBX to the FOXO3-DBD protein was analyzed by FAM-EMSA. 1 µM recombinant FOXO3 and 100 nM fluorescence-labeled FoxP3- or FoxP3-mutated oligonucleotides were incubated with increasing concentrations of CBX for 30 min at room temperature. In the sample marked with (-) no FOXO3-DBD protein was added. Densitometric analysis of the FOXO3-DBD/FoxP3 complex signal was done with the ImageJ 1.48 software. The untreated control was set as 100%. **d** FAM-EMSA was performed using 30 µg whole-cell extracts of SH-EP/FOXO3 cells treated with 50 nM 4OHT to activate FOXO3(A3)ERtm in combination with indicated concentrations of CBX for four hours. The whole-cell extracts were incubated with 100 nM fluorescence-labeled FoxP3- or FoxP3-mutated oligonucleotides for 30 min at room temperature. As indicated, cells were cultivated under standard (10% FCS) or serum starved (0.1% FCS) conditions. Equal loading of cellular protein extracts was ensured by immunoblot analysis with the anti-GAPDH antibody. Densitometric analysis of the FOXO3-DBD/oligonucleotide complex signal was done with the ImageJ 1.48 software. The controls without CBX treatment were set as 100%

To determine the binding properties of CBX to the FOXO3-DBD protein, we first analyzed the dissociation constant (K_d_) of the FOXO3-DBD protein/IRE-FAM oligonucleotide interaction. Therefore, we used a constant concentration of the IRE-FAM oligonucleotide and titrated the recombinant FOXO3-DBD protein at increasing concentrations. We measured a K_d_ value of 53.4 nM in respect to our assay conditions (Fig. 2a).

To determine the IC50 of CBX, a competitive binding experiment with constant concentrations of the FOXO3-DBD protein and the FAM-labeled oligonucleotide was performed. CBX was titrated with increasing concentration and the IC50-value of 29.7 µM was calculated by nonlinear least-square analysis. The binding affinity (K_i_)-value of CBX was assessed by the equation of Nikolovska–Coleska [34] based on the measured IC50-values, the K_d_-value of the protein/oligonucleotide complex (FOXO3-DBD/IRE-FAM oligonucleotide), the concentration of the FOXO3-DBD protein (25 nM), as well as the IRE-FAM oligonucleotide (5 nM) used in the assay. We determined a K_*i*_-value of 19.0 µM for CBX at our assay conditions (Fig. 2b).

To validate the interaction between CBX and recombinant FOXO3-DBD protein, we performed a fluorescence-based electrophoretic mobility shift assay (FAM-EMSA) using the FOXO-consensus sequence present in the FoxP3 promoter (FoxP3 oligonucleotide) [35, 36] that binds with high affinity to the FOXO3-DBD protein resulting in a detectable band shift (Fig. 2c). Incubation of the FOXO3-DBD protein/FoxP3-FAM oligonucleotide complex with increasing concentrations of CBX for 30 min resulted in a dose-dependent reduction of the band shift. Sixty micromolar of CBX completely prevented binding of the FOXO3-DBD to the oligonucleotide. Specific binding of the FoxP3 oligonucleotide to the FOXO3-DBD protein was validated by using mutant FoxP3 oligonucleotides [35] (Fig. 2c).

To assess whether CBX also inhibits binding of the endogenous FOXO3 to the FoxP3 oligonucleotide in NB cells a FAM-EMSA using whole-cell lysates of SH-EP/FOXO3 cells treated with indicated concentrations of CBX for four hours, was performed. SH-EP/FOXO3 cells stably express a 4-hydroxy-tamoxifen-inducible (4OHT), PKB-phosphorylation-independent FOXO3(A3) estrogen receptor ligand binding domain (ERtm) transgene. Activation of FOXO3 by 4OHT induces apoptosis in the stroma-like SH-EP/FOXO3 cells [20]. Incubation of the cell lysates with the FAM-labeled FoxP3 oligonucleotide induced the formation of the FOXO3/FoxP3-FAM complex, which was repressed by CBX treatment in a dose-dependent manner. FAM-EMSA with cell lysates using mutated FoxP3 oligonucleotides showed no complex formation, indicating specific binding of the FoxP3 oligonucleotides to FOXO3. Equal loading of cellular protein extracts was ensured by immunoblot using 30 µg of cellular protein extracts and the anti-GAPDH antibody (Fig. 2d).

In the cell-based FAM-EMSA up to 120–155 µM of CBX were needed to inhibit binding of FOXO3 to the oligonucleotide (Fig. 2d), compared with 60–80 µM CBX in the non-cell-based assay (Fig. 2c). This discrepancy is likely due to binding of CBX to albumin in the used cell culture media [37]. Using media with reduced amounts of fetal calf serum (medium supplemented with 0.1% FCS), 80 µM CBX were sufficient to halve the signal (Fig. 2d), indicating that higher concentrations of CBX are needed in cell culture experiments when standard medium with 10% FCS is used.

### CBX inhibits the transcriptional activity of FOXO3 in NB cells

**Fig. 3:**
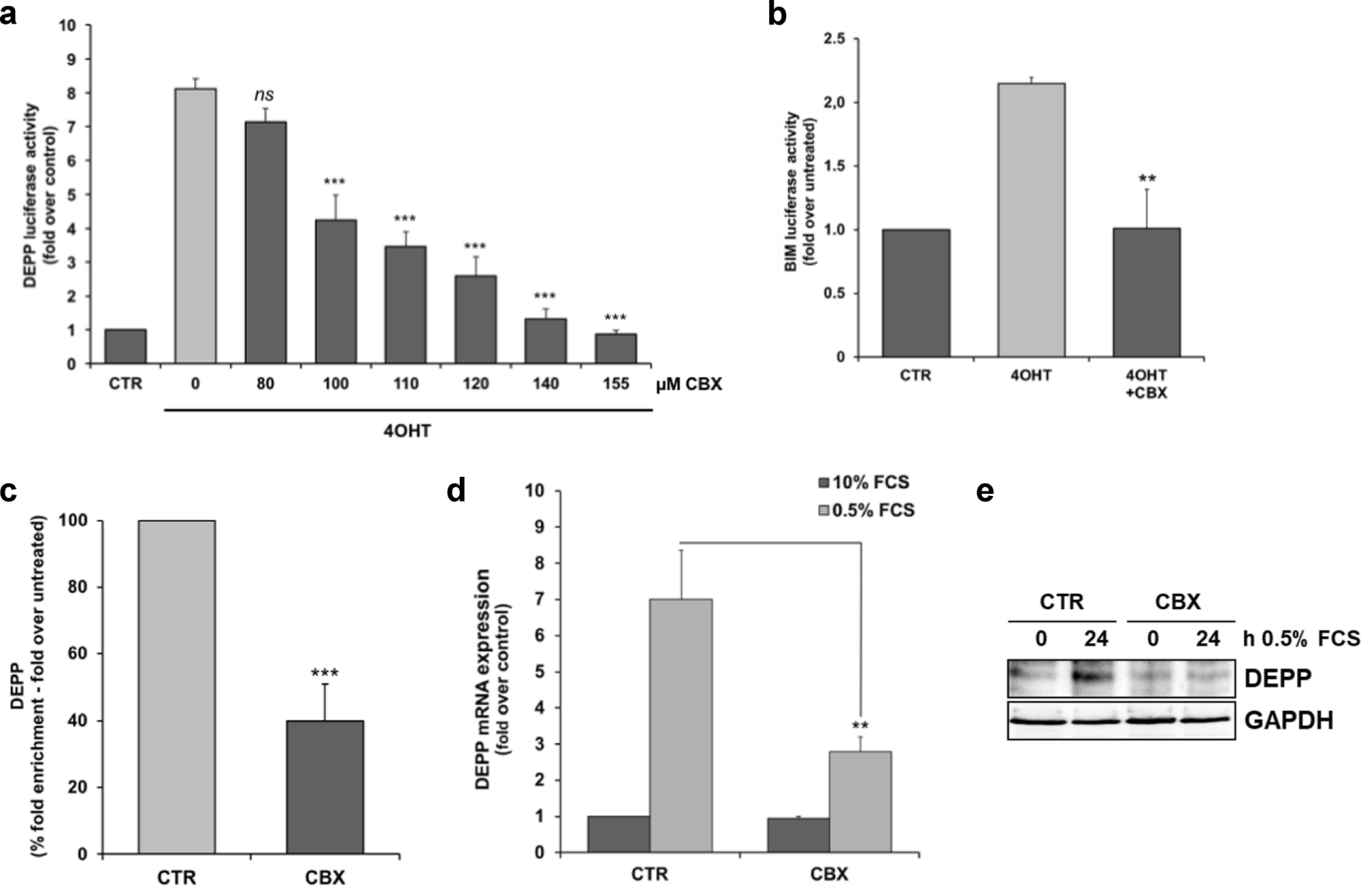
CBX interacts with the FOXO3-DBD and inhibits its transcriptional activity in NB cells. **a** The DEPP-promoter reporter plasmid was transfected into SH-EP/FOXO3 cells and a luciferase activity assay was performed. The cells were treated with 20 nM 4OHT to activate ectopic FOXO3(A3)ERtm and incubated with the indicated concentrations of CBX for eight hours. Direct binding of FOXO3 to the DEPP-promoter activates this reporter system as described before [40]. The increase of the luciferase signal was calculated as fold over untreated controls. Shown are mean values ± s.e.m. of three independent experiments; statistical analysis was done with the Student’s unpaired *t* test; ***p < 0.01 compared with 4OHT-treated cells. **b** A luciferase activity assay was performed in SH-EP/FOXO3 cells transfected with a BIM-promoter reporter plasmid. The cells were treated with 50 nM 4OHT alone or in combination with 140 µM CBX for eight hours. The increase of the luciferase signal was calculated as fold over untreated controls. Shown are mean values ± s.e.m. of three independent experiments; statistical analysis was done with the Student’s unpaired *t* test; **P < 0.025 compared with 4OHT-treated cells. **c** ChIP analyses were performed in SH-EP/FOXO3 cells treated with 100 nM 4OHT alone or in combination with 120 µM CBX for three hours. Binding of FOXO3 to the promoter region of DEPP was assessed by quantitative RT-PCR. Shown are mean values ± s.e.m. of three independent experiments. Statistical analysis was done with the Student’s unpaired *t* test; ***P < 0.01 compared with the 4OHT-treated control (%). **d**, **e** The impact of CBX treatment on FOXO3-dependent induction of DEPP expression in serum starved cells was analyzed by quantitative RT-PCR (**d**) and by immunoblot analyses (**e**). For quantitative RT-PCR analysis of DEPP expression, serum starved SH-EP cells (0.5% FCS) were treated with 80 µM CBX for 24 hours. Shown are means ± s.e.m. of three independent experiments. Statistical analysis was done with the Student’s unpaired *t* test, **P < 0.025 compared with the serum starved control. For immunoblot analyses, SH-EP cells were cultivated under serum starvation conditions (0.5% FCS) and incubated with 80 µM CBX for 24 hours. GAPDH served as loading control

We identified the reactive oxygen species (ROS) regulating decidual-protein-induced-by-progesterone (DEPP/DEPP1/C10orf10) and the pro-apoptotic BH3-only protein B-cell-lymphoma-gene-2-like-11 (BCL2L11/BIM) as direct transcriptional target genes of FOXO3 in SH-EP/FOXO3 cells [20, 38]. To analyze the inhibitory capacity of CBX on the transcriptional activity of FOXO3, we performed DEPP- and BIM-specific luciferase activity assays [38, 39] in SH-EP/FOXO3 cells. Activation of the ectopic FOXO3(A3) ERtm construct by 4OHT elevated the DEPP-luciferase activity ~8.5-fold (Fig. 3a) and the BIM-luciferase activity ~2.3-fold (Fig. 3b) compared with untreated cells, respectively. Treatment with CBX efficiently repressed the FOXO3-mediated DEPP-promoter activity in a dose-dependent manner (Fig. 3a). A concentration of 100 µM CBX was sufficient to significantly (*P* < 0.01) repress the FOXO3-mediated DEPP-promoter activity (Fig. 3a). Treatment with 140 µM CBX reduced the DEPP- and the BIM-promoter activity to control level, respectively (Fig. 3a, b). Of note, the CBX concentrations needed to repress the FOXO3-transcriptional activity are in line with the concentrations determined in the FAM-EMSA experiments (Fig. 2c, d). To further investigate whether CBX inhibits binding of FOXO3 to the DEPP promoter in living cells, we performed chromatin immunoprecipitation (ChIP) analyses. In concordance, ChIP analyses demonstrated that CBX abrogates binding of FOXO3 to the DEPP-promoter in SH-EP/FOXO3 cells cultivated in presence of 100 nM 4OHT, further supporting the notion that CBX directly suppresses the transcriptional activity of FOXO3 (Fig. 3c).

As FOXO3 is essential for DEPP-induction during growth factor withdrawal [38], SH-EP cells were cultivated under serum starvation conditions (0.5% FCS) in absence and presence of CBX. CBX efficiently inhibited the DEPP mRNA and protein expression triggered by endogenous FOXO3 (Fig. 3d, e). In previous studies we could demonstrate that the chemotherapeutic agent etoposide activates FOXO3 [4] and induces the expression of the FOXO3 target DEPP [2]. Etoposide-triggered DEPP mRNA and protein expression was markedly abrogated by CBX treatment in SH-EP cells (Supplementary Fig. S5a, b), supporting the concept that CBX represses the transcriptional activity of endogenous FOXO3 in NB cells.

In summary, these findings indicate that CBX binds to the FOXO3-DBD and silences thereby the FOXO3-triggered transcriptional target gene regulation in NB cells.

### CBX inhibits FOXO3-mediated cell death in stroma-like, low-stage NB cells

**Fig. 4:**
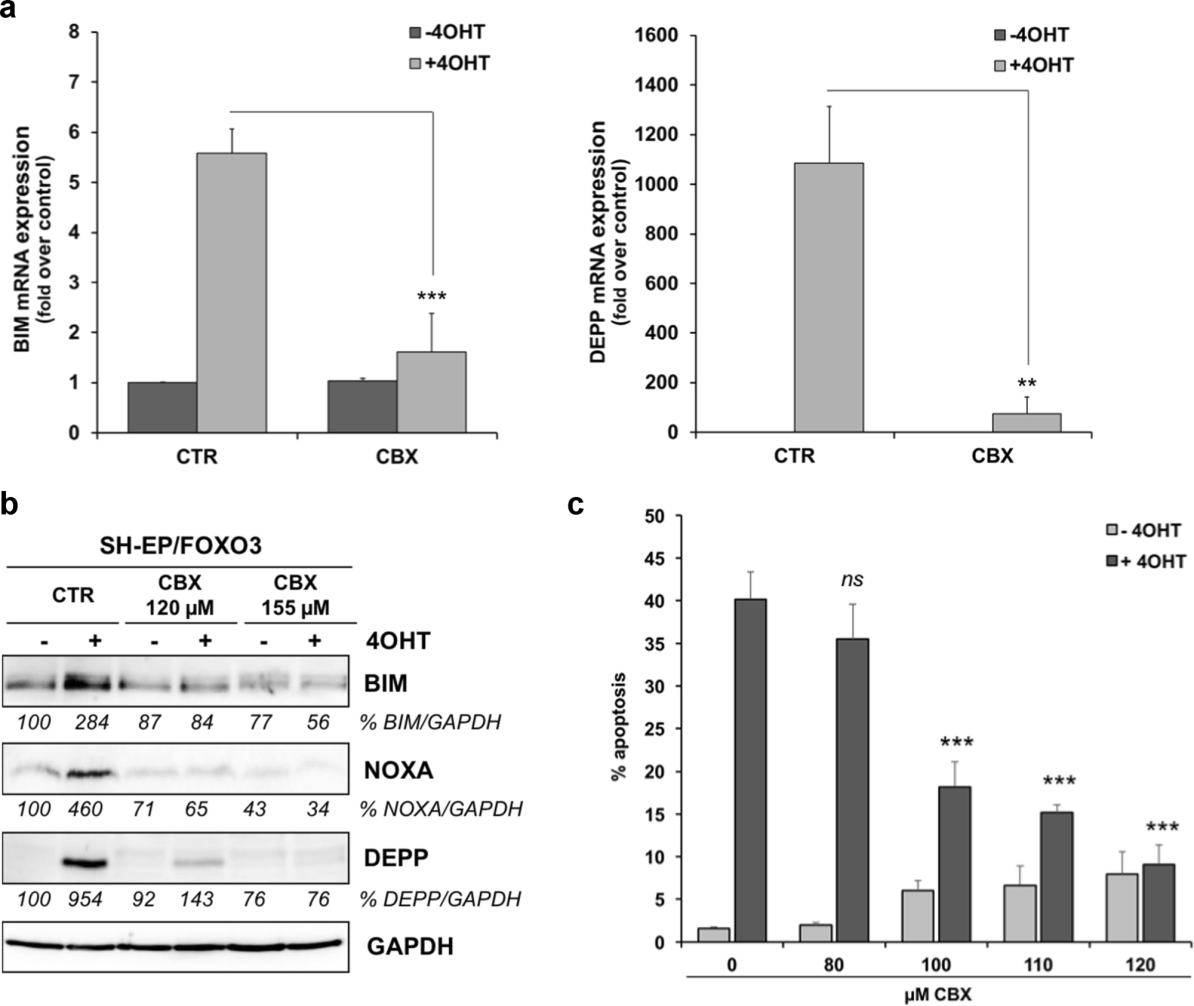
FOXO3-dependent apoptosis is suppressed by CBX in low-stage NB cells. **a** Quantitative RT-PCR analyses of BIM and DEPP expression in SH-EP/FOXO3 cells treated with 20 nM 4OHT in combination with 120 µM CBX for six hours. Shown are means ± s.e.m. of three independent experiments. Statistical analysis was done with the Student’s unpaired *t* test; **P < 0.025, ***P < 0.01 compared with the corresponding control. **b** Immunoblot analyses of BIM, NOXA, and DEPP expression in SH-EP/FOXO3 cells treated with 50 nM 4OHT and with indicated concentrations of CBX for eight hours. GAPDH served as loading control. Densitometric analyses of BIM, NOXA, and DEPP expression relative to GAPDH were done with the ImageJ 1.48 software. Untreated cells were set as 100%. **c** SH-EP/FOXO3 cells were treated with 20 nM 4OHT and with indicated concentrations of CBX for 48 hours. PI-FACS analyses were performed to detect apoptotic cells. Shown are mean values ± s.e.m. of three independent experiments. Statistical analysis was done with the Student’s unpaired *t* test; ***P < 0.01 compared with the 4OHT-treated control

FOXO3 triggers the expression of phorbol-12-myristate-13-acetate-induced-protein-1 (PMAIP1/NOXA), BIM, and DEPP, causing an increase in cellular ROS steady-state levels and consequently induction of apoptosis as well as autophagy in low-stage SH-EP/FOXO3 cells [20, 38, 40]. By quantitative RT-PCR analyses of SH-EP/FOXO3 cells treated with 4OHT we found that the FOXO3-triggered expression of both, BIM and DEPP, was markedly repressed by treatment with 120 µM CBX (Fig. 4a). In line, FOXO3-induced BIM, DEPP, as well as NOXA protein expression was dose dependently repressed by CBX treatment (Fig. 4b).

FOXO3-induced BIM and DEPP expression is associated with biphasic ROS accumulation that triggers apoptosis in SH-EP/FOXO3 cells [38, 41]. Consistent with inhibition of BIM, DEPP, and NOXA expression, CBX repressed the FOXO3-dependend ROS induction (data not shown). FOXO3-triggered apoptotic cell death was significantly diminished by CBX treatment in a dose-dependent manner (Fig. 4c). A concentration of 100 µM CBX was sufficient to significantly (P < 0.01) reduce the FOXO3-mediated cell death. Incubation with 120 µM CBX lowered the FOXO3-triggered apoptosis rate to <10% dead cells (Fig. 4c). Of note, treatment with enoxolone, that showed no interaction with the FOXO3-DBD (Supplementary Fig. S4b), did not repress FOXO3-triggered apoptosis in SH-EP/FOXO3 cells (Supplementary Fig. S4d). Together, these data demonstrate that the compound CBX silences the transcriptional activity of FOXO3 and thereby abrogates FOXO3-mediated cell death in “FOXO3-sensitive” low-stage NB cells.

### CBX abolishes FOXO3-triggered SESN3 expression and associated clonogenic cell survival in high-stage NB cells

In NB nuclear FOXO3 predicts adverse clinical outcome, promotes tumor angiogenesis [18], and mediates chemoprotection in the high-stage NB cell lines NB1, NB4, and NB8. In these cells FOXO3-induced chemoprotection depends on wild-type TP53 and SESN3 expression [19].

**Fig. 5:**
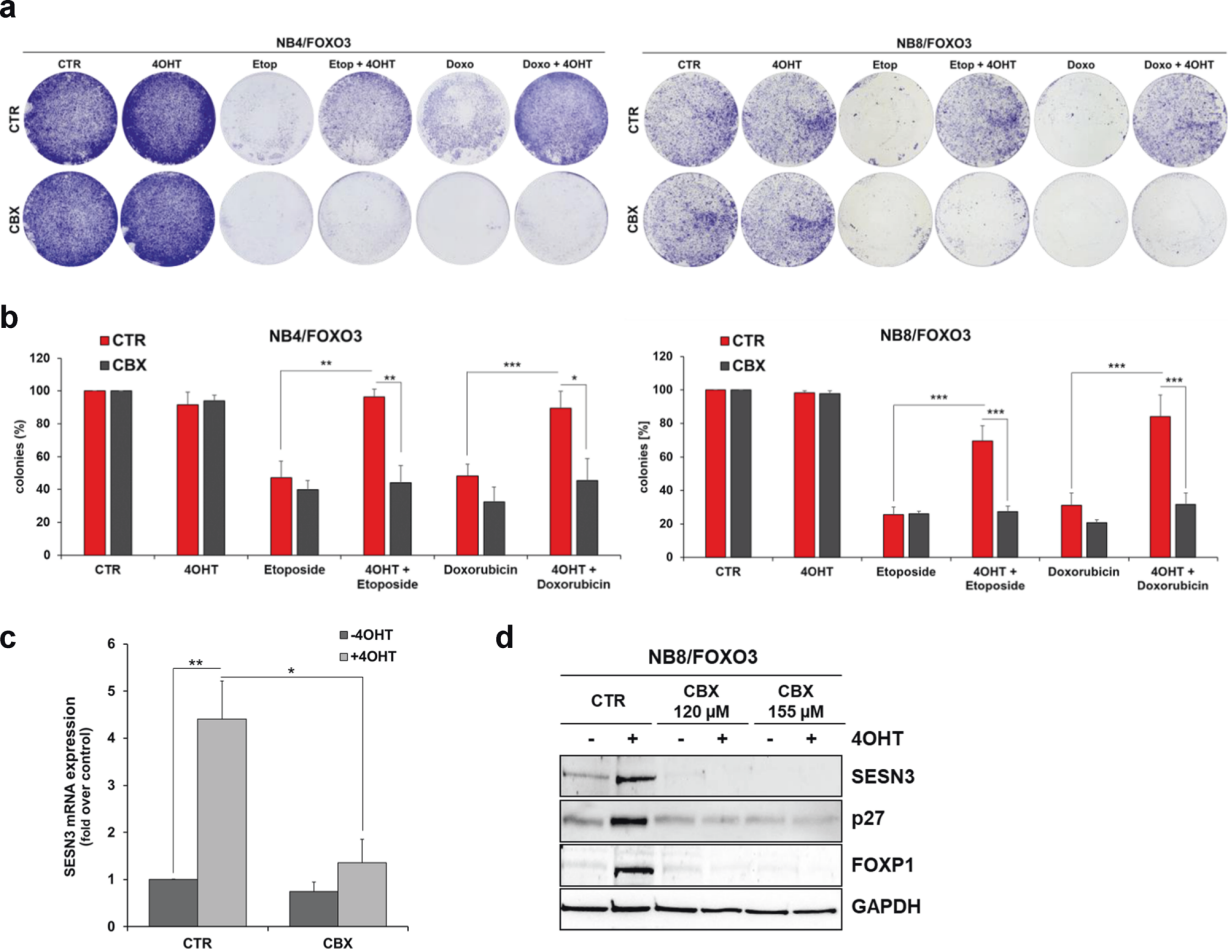
CBX inhibits FOXO3-mediated SESN3 expression and associated chemoprotection in high-stage NB cells. **a** Clonogenic survival of NB4/FOXO3 and NB8/FOXO3 cells was analyzed by CFA. The cells were treated for 72 hours with 50 nM 4OHT and 80 µM CBX alone and for further 72 hours with etoposide and doxorubicin in combination. A concentration of 0.1 µg/ml etoposide or 0.01 µg/ml doxorubicin were added to NB4/FOXO3 cells and 0.8 µg/ml etoposide or 0.08 µg/ml doxorubicin to NB8/FOXO3 cells. Colonies were stained with crystal violet. **b** Quantification of the CFA was performed by photometric measurement after discoloration with 0.5% SDS in 50% ethanol. Shown are means ± s.e.m. of at least three independent experiments; *P < 0.05, **P < 0.025, ***P < 0.01. **c** NB8/FOXO3 cells were treated with 100 nM 4OHT alone or in combination with 120 µM CBX for six hours and quantitative RT-PCR of SESN3 expression was performed. Shown are mean values ± s.e.m. of three independent experiments. Statistical analyses were done with the Student’s unpaired *t* test; *P < 0.05, **P < 0.025 compared with controls. **d** Immunoblot analyses of SESN3, P27KIP1, and FOXP1 expression in NB8/FOXO3 cells treated with 50 nM 4OHT and the indicated concentrations of CBX for 24 hours. GAPDH served as loading control

Here we show that the FOXO3-inhibitory compound CBX abrogates the chemo-protective effect of FOXO3 in these high-stage NB cell lines. By colony formation assay (CFA) we demonstrate that the FOXO3-mediated pro-survival phenotype of etoposide and doxorubicin treated NB4/FOXO3 and NB8/FOXO3 cells was significantly repressed by CBX treatment (Fig. 5a, b). Activation of ectopic FOXO3 by 4OHT significantly increased the colony forming capacity of both high-stage NB cell lines. FOXO3 inhibition by 80 µM CBX efficiently repressed the FOXO3-triggered increase in clonogenic cell survival (Fig. 5a, b).

This chemo-protective function of FOXO3 is mediated by SESN3 induction in these high-stage NB cells [19]. CBX treatment prevented FOXO3-mediated transcriptional expression of SESN3 in NB8/FOXO3 cells on mRNA (Fig. 5c) and protein level (Fig. 5d).

In addition, CBX abrogated the FOXO3-mediated induction of the cell cycle regulator CDKN1B/P27KIP1, a *bona-fide* target of FOXO3 [42] and of the forkhead box P1 (FOXP1) protein, an essential modulator of FOXO3-triggered cellular survival [43], in NB8/FOXO3 cells (Fig. 5d). This suggests that the transcriptional inhibition of the pro-survival FOXO3 target genes SESN3 and FOXP1 by CBX treatment revokes FOXO3-triggered chemoprotection in high-stage NB.

### Chemo-sensitizing effects of CBX on high-stage NB cell lines

To analyze potential chemo-sensitizing effects of CBX on high-stage NB cells we performed PI-FACS analyses of NB1 and NB8 cells treated with etoposide, doxorubicin, and CBX alone or in combination for 72 hours.

**Fig. 6:**
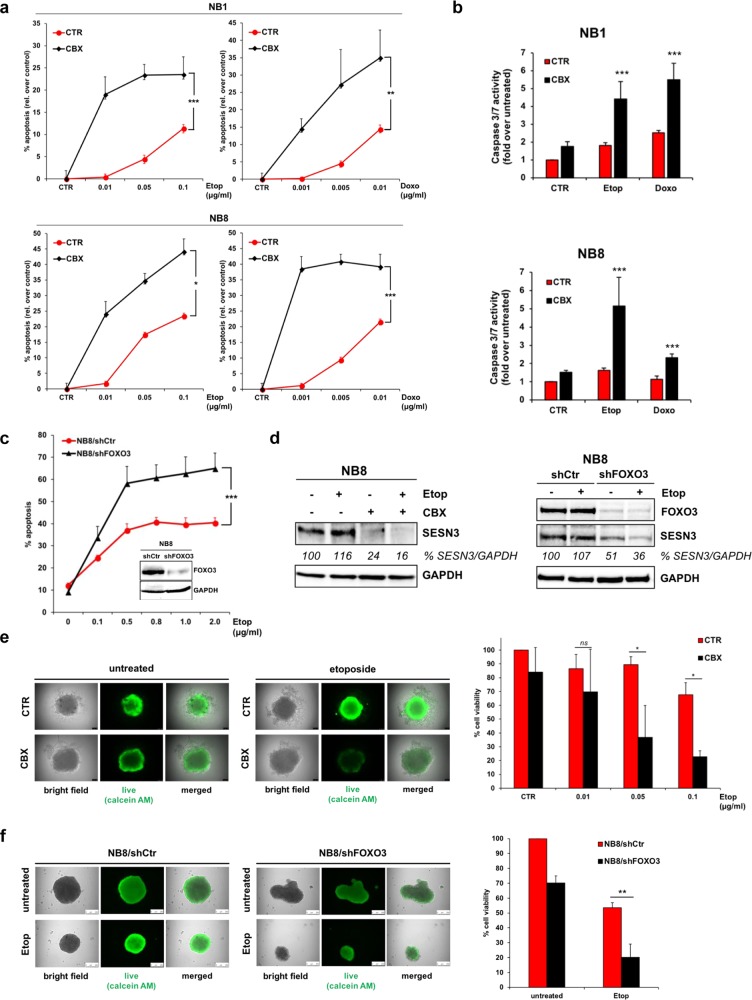
Inhibition of FOXO3 by CBX sensitizes high-stage NB cells to chemotherapy. **a** The high-stage NB cell lines NB1 and NB8 were treated with the indicated concentrations of etoposide and doxorubicin in combination with 120 µM CBX for 72 hours. PI-FACS analyses were performed to detect apoptotic cells. Shown are mean values ± s.e.m. of three independent experiments. Statistical analysis was done with the Student’s unpaired *t* test; *P < 0.05, **P < 0.025, ***P < 0.01 compared with corresponding controls. **b** The caspase 3/7 activity assay was performed in NB1 and NB8 cells treated with 0.1 µg/ml etoposide or 0.01 µg/ml doxorubicin alone or in combination with 80 µM CBX for 48 hours. Shown are the means ± s.e.m. of three independent experiments, statistical analysis was done with the Student’s unpaired *t* test, ***P < 0.01 compared with corresponding controls. **c** Immunoblot analyses of FOXO3 expression in NB8/shCtr and NB8/shFOXO3 cells. GAPDH served as loading control. PI-FACS analyses were performed to detect apoptotic cells in NB8/shCtr and NB8/shFOXO3 cells treated with the indicated concentrations of etoposide for 72 hours. Shown are mean values ± s.e.m. of three independent experiments. Statistical analysis was done with the Student’s unpaired *t* test; ***P < 0.01 between drug-treated cell lines. **d** Immunoblot analyses of SESN3 expression in NB8 cells treated with 10 µg/ml etoposide alone or in combination with 120 µM CBX for four hours (left panel) and in NB8/shCtr and NB8/shFOXO3 cells treated with 10 µg/ml etoposide for four hours (right panel). GAPDH served as loading control. Densitometric analysis of SESN3 expression relative to GAPDH was done with the ImageJ 1.48 software. Untreated cells were set as 100%. **e** Representative images of NB8 spheroids formed by th**e** hanging-drop technique for 96 hours. The cells were treated with 0.1 µg/ml etoposide in combination with 120 µM CBX for another 96 hours. Viable cells were stained with 2 µM calcein-AM for two hours at 37 °C. Quantification of viable cells in NB8 spheroids was done by the CellTiter-Glo 3D® cell viability assay. The cells were treated with indicated concentrations of etoposide in combination with 120 µM CBX for 96 hours. Shown are mean values ± s.e.m. of three independent experiments. Statistical analysis was done with the Student’s unpaired *t* test; *P < 0.05 between ±CBX treatment. **f** Representative images of NB8/shCtr and NB8/shFOXO3 spheroids formed by the hanging-drop technique for 96 hours treated for further 96 hours with 0.1 µg/ml etoposide. Viable cells were stained with 2 µM calcein-AM for two hours at 37 °C. Cell viability of NB8/shCtr and NB8/shFOXO3 cells was analyzed by the CellTiter-Glo 3D® cell viability assay. The spheroids were treated with 0.1 µg/ml etoposide for 96 hours. Shown are mean values ± s.e.m. of three independent experiments. Statistical analysis was done with the Student’s unpaired *t* test; **P < 0.025 between drug-treated cell lines

CBX sensitized these cell lines to chemotherapy as apoptosis induction was significantly elevated due to treatment with 120 µM CBX (Fig. 6a). In concordance with these findings, the caspase 3/7 activity significantly increased due to FOXO3 inhibition by CBX in etoposide and doxorubicin treated NB1 and NB8 cells (Fig. 6b). In line with our previous studies [19], stable expression of FOXO3-specific shRNA significantly sensitized high-stage NB8 cells to etoposide treatment (Fig. 6c). Also, the expression of the ROS detoxifying protein SESN3 was markedly repressed due to FOXO3 inhibition by CBX, as well as by FOXO3-knockdown in etoposide-treated NB8 cells (Fig. 6d).

The pro-survival phenotype of FOXO3 is also visible in 3D-tumor-spheroids, as FOXO3-activation significantly increases spheroid size and viability [19]. The beneficial effect of FOXO3 inhibition by CBX was also evident in 3D-tumor-spheroids derived from high-stage NB8 cells. FOXO3-silencing by 120 µM CBX in etoposide treated 3D-tumor-spheroids significantly impaired spheroid viability as measured by live-cell calcein-AM staining and by quantification of the cell viability by ATP content (Fig. 6e). FOXO3 expression seems to be necessary for the generation of uniform shaped 3D-tumor-spheroids as in this setting FOXO3-knockdown resulted *per se* in reduced spheroid size. Also, FOXO3-knockdown significantly sensitized NB8 cells to chemotherapy as measured by ATP content (Fig. 6f).

Together, the results suggest that targeting the transcriptional activity of FOXO3 by CBX efficiently abrogates the pro-survival function of FOXO3 and sensitizes high-stage NB cells to chemotherapy in 2D and 3D cell culture models.

## Discussion

As several studies point out the potential oncogenic properties of FOXO3 in different cancer types, pharmacological inhibition of FOXO3 is of great interest for novel therapeutic interventions in these cancers [4–17]. In accordance, we could demonstrate that nuclear FOXO3 promotes tumor angiogenesis in vivo [18] and chemoprotection in vitro [19] in aggressive NB. Therefore, this study was designed to identify novel clinically-approved FOXO3 inhibitors by a drug-repositioning strategy. By FPA-screening of the Prestwick Chemical Library^®^, consisting of 1120 FDA-approved drugs, we identified CBX as potential FOXO3-inhibitory compound (Fig. 1) with a FOXO3-DBD specific binding affinity (Ki) value of 19.0 µM (Fig. 2b).

CBX represents a glycyrrhetinic acid derivative, which has been used for the treatment of gastritis and peptic ulcer [44]. Besides its role as 11β-hydroxysteroid dehydrogenase (11β-HSD) inhibitor and modulator of the glucocorticoid metabolism, CBX also blocks gap junction communication [45]. Concerning a possible use of CBX in cancer, it has been shown that CBX inhibits the expression of the apoptosis-inhibitor-protein BIRC5/survivin and thereby triggers apoptosis in K562 leukemic cells [46] and enhances TRAIL-induced apoptosis in human glioma [47]. Regarding its function as a gap junction inhibitor, particularly as a potent inhibitor of the pannexin PANX1, CBX represents a promising molecule to treat different forms of cancer, as the majority of reports point towards a tumor promoting effect of PANX1 expression, especially in late stage or advanced cancer and in metastasis [48].

In the present study we show that CBX targets the FOXO family members FOXO1, FOXO3, FOXO4, and FOXO6 (Supplementary Fig. S3b) in a dose-dependent manner via binding to their highly conserved forkhead DBD [32]. However, as exclusively FOXO3 is expressed in the analyzed high-stage NB cell lines (Supplementary Fig. S3c), this study was designed to investigate the efficacy of CBX to inhibit FOXO3 in order to overcome chemoprotection in neuronal tumor cells.

We demonstrate that CBX interferes with the FOXO3-DBD-DNA binding and thereby silences FOXO3-transcriptional activity. Interaction of CBX with the FOXO3-DBD was validated by FPA (Fig. 1d) and EMSA (Fig. 2c, d). In vitro studies were initially done in “FOXO3-sensitive” SH-EP/FOXO3 cells expressing a 4OHT, PKB-phosphorylation-independent FOXO3(A3)ERtm transgene [20] to assess the inhibitory effects of CBX on FOXO3 activity. We found that FOXO3-mediated activation of the DEPP-promoter, a direct transcriptional target gene of FOXO3 in NB cells [38], was repressed by CBX treatment in a dose-dependent manner as shown by a DEPP-promoter reporter assay (Fig. 3a) and by a chromatin immunoprecipitation experiment (Fig. 3c). In line, the promoter activity of the FOXO3 target gene BIM [20] was efficiently suppressed by CBX incubation (Fig. 3b).

Importantly, CBX treatment also abrogated starvation-triggered induction of DEPP expression that is mediated by endogenous FOXO3 in SH-EP cells (Fig. 3d, e) [38]. The chemotherapeutic agent etoposide activates FOXO3 [4] and induces the expression of its target DEPP [2] in NB. Etoposide-mediated DEPP expression was efficiently abrogated by CBX treatment, supporting the notion that CBX represses the transcriptional activity of endogenous FOXO3 (Supplementary Fig. S5).

**Fig. 7:**
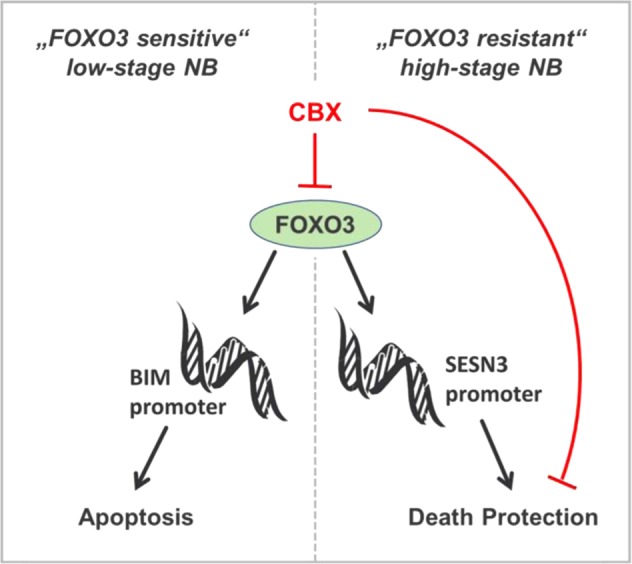
Proposed mechanism how silencing of the FOXO3-transcriptional activity by CBX affects cell death in NB cells. In low-stage “FOXO3-sensitive” NB cells FOXO3 inhibition prevents BIM induction and thus FOXO3-dependent cell death. On the other hand, in the “FOXO3-resistant” high-stage derived NB cells, FOXO3-mediated death protection by SESN3 expression is abrogated by CBX treatment. Consequently FOXO3 inhibition by CBX sensitizes the resistant NB cells to chemotherapy

CBX silenced FOXO3-mediated gene expression of NOXA, BIM, and DEPP on RNA (Fig. 4a) and protein level (Fig. 4b) and consequently inhibited FOXO3-triggered apoptosis in low-stage “FOXO3-sensitive” SH-EP/FOXO3 cells (Fig. 4c and 7). Enoxolone that did not interact with the FOXO3-DBD as demonstrated by FPA (Supplementary Fig. S4b), had no effect on FOXO3-triggered apoptosis in SH-EP/FOXO3 cells (Supplementary Fig. S4d). Docking studies suggested that this is due to the lack of the hemisuccinate carboxylate in enoxolone, which prevents further ionic interactions with basic residues of the FOXO3-DBD that are also involved in charged interactions with the DNA backbone (Supplementary Fig. S4c).

Of note, the protective effect of CBX-mediated FOXO3 inhibition on neuronal cells might also be of great interest in regard to neurodegenerative diseases. Targeting FOXO3 may represent a novel strategy for the development of therapies against Alzheimer’s disease (AD) and cognitive impairment, as it plays a crucial role in promoting neuronal cell death by upregulating BIM during glutamate-associated excitotoxicity and amyloid-β peptide (Aβ) toxicity, both hallmarks of AD [49–55]. Suppression of FOXO3 also confers neuroprotective effects after traumatic brain injury [56] and against ischemic injury [57–59]. Remarkably, CBX has been described to improve cognitive function in healthy elderly men and type 2 diabetics [60] and to be neuroprotective during brain damage following intracerebral hemorrhage [61]. CBX further suppresses disease progression in mouse models of amyotrophic lateral sclerosis and AD by inhibiting glutamate release from activated microglia without producing notable toxicity [62]. As NB cells are the most frequently utilized models in neurodegenerative research [63], the potential beneficial effects of FOXO3 inhibition by CBX on neurotoxicity could be a promising starting point for further studies.

We recently demonstrated that FOXO3 regulates SESN3 expression and thereby mediates chemoprotection in aggressive high-stage NB [19]. Notably, we found that FOXO3 inhibition by CBX treatment efficiently repressed FOXO3-triggered SESN3 expression in the high-stage NB8 cell line on mRNA and protein level (Fig. 5c, d). CBX also abrogated the FOXO3-mediated induction of the cell cycle regulator CDKN1B/P27KIP1, a *bona-fide* target gene of FOXO3 (Fig. 5d) [42]. The expression of the FOXO3-transcriptional target gene FOXP1 was markedly inhibited by CBX treatment in NB8/FOXO3 cells (Fig. 5d), which is an important finding as Boxtel et al. demonstrated that FOXP1 represents an essential modulator of FOXO-induced transcription, promoting cellular survival [43]. In concordance with these findings, we found that FOXO3 inhibition by CBX efficiently repressed FOXO3-mediated clonogenic survival in NB8/FOXO3 and in NB4/FOXO3 cells cultivated in the presence of the chemotherapeutic drugs etoposide or doxorubicin (Fig. 5a, b).

FOXO3 inhibition by CBX significantly sensitized high-stage NB cells to etoposide and doxorubicin treatment as demonstrated by increased cellular apoptosis (Fig. 6a) and elevated caspase 3/7 activity (Fig. 6b). This chemo-sensitizing effect of FOXO3 inhibition was also visible in NB8 cells by stable expression of FOXO3-specific shRNA (Fig. 6c). In line, CBX treatment as well as FOXO3-knockdown markedly repressed SESN3 expression in etoposide treated NB8 cells (Fig. 6d).

The chemo-sensitizing effect of FOXO3 inhibition was even more evident in a 3D cell culture model. In tumor-spheroids derived from high-stage NB8 cells, viability was markedly repressed when etoposide was applied in combination with CBX (Fig. 6e). FOXO3-knockdown cells only formed loose aggregates with smaller size compared with the uniform shape and consistent size in 3D cultivated NB8-shCtr spheroids (Fig. 6f), showing that FOXO3 is necessary for compact spheroid formation. In line with the beneficial effect of FOXO3 inhibition by CBX, FOXO3-knockdown abrogated chemoresistance in 3D spheroids derived from high-stage NB8 cells (Fig. 6f). One explanation for this effect of FOXO3 inhibition in the 3D culture model is, that FOXO3 activity is elevated in spheroids derived from neuronal tumor cells *per se*, which initiates drug resistance, possibly through FOXO3-triggered activation of autophagy [64]. In concordance, our own studies describe FOXO3 as a key regulator of autophagic flux and demonstrate that inhibition of FOXO3-mediated autophagy sensitizes NB cells to chemotherapy [40].

Regarding the potential clinical use and therapeutic efficacy of CBX the pharmacologically relevant plasma concentrations have to be considered. Our in vitro studies revealed that 80–140 µM CBX are needed to efficiently silence FOXO3-transcriptional activity. As shown by EMSA (Fig. 2d), the relatively high concentrations of CBX are needed due to binding to BSA, which is in concordance with the literature [37]. Importantly, CBX is rapidly absorbed in vivo following oral administration of an aqueous solution of the sodium salt to patients and attains high blood plasma concentrations of up to 175 µM without severe side effects [65] – this exceeds the concentration at which we observed FOXO3 inhibition, suggesting its potential clinical application.

To date only FOXO1 and FOXO4 specific inhibitors have been developed. The FOXO1 specific small molecular compound AS1842856 has been analyzed as therapeutic drug for the treatment of type 2 diabetes in mice, and has been tested in regard of pulmonary hypertension and bone development. FOXO1 inhibition by AS1842856 suppresses adipogenesis and diminishes vascular insulin resistance in human obesity [66–70]. In HER2 positive breast cancer cells AS1842856 restores Lapatinib sensitivity by reducing the FOXO1-mediated MYC upregulation [71] and in ovarian cancer cells AS1842856 prevents the FOXO1-induced senescence by inhibiting the progestin-induced p21 expression [72]. Furthermore, two peptides that mimic the FOXO1-IQGAP1 and the FOXO4-p53 interaction have so far been reported [73, 74]. The FOXO1-derived peptide inhibitor overcomes drug resistance against the PI3K and paclitaxel in prostate cancer cells [73]. The FOXO4 peptide causes p53 nuclear exclusion and apoptosis in senescent cells after severe DNA damage and restores fitness in fast aging mice [74]. This FOXO4 mimicking peptide has until now not been tested in cancer cells. Our own data show that CBX interacts also with the FOXO family members FOXO1, FOXO4, and FOXO6 (Supplementary Fig. S3b). The alleged impact of CBX on other FOXOs could be a promising starting point for further research.

In summary, our data describe the first FDA-approved small molecular weight inhibitor of FOXO transcription factors. CBX interacts with the FOXO3-DBD and silences thereby its transcriptional activity. Hence, FOXO3-mediated chemoprotection is efficiently abrogated in high-stage NB cells by CBX treatment (Fig. 7). Especially, CBX exerts its chemo-sensitizing effects in pharmacologically relevant concentrations and might therefore be a promising novel candidate for the treatment of therapy-resistant high-stage NB patients. Clearly, future more in-depth studies are needed to define the clinical applications of FOXO3 inhibition by CBX in high-stage NB, in other “FOXO-resistant” cancers, or even in neurodegenerative diseases.

## Materials and methods

### Cell lines, culture conditions and reagents

The NB cell lines STA-NB1, STA-NB4, and STA-NB8, provided by the St. Anna children’s hospital, Vienna, Austria, are termed NB1, NB4, and NB8, respectively [75]. The NB cell line SH-EP was a kind gift of N. Gross, Lausanne, Switzerland [76]. 2D and 3D cultivation of the cell lines was done in RPMI1640 medium (Lonza, Basel, Switzerland) supplemented with 10% fetal calf serum (Sigma-Aldrich, Vienna, Austria), 100 U/ml penicillin, 100 µg/ml streptomycin and 2 mM L-glutamine (Lonza, Basel, Switzerland) at 37 °C and 5% CO_2_ in saturated humidity. Phoenix^TM^ packaging cells for helper-free production of amphotropic retroviruses [77] and HEK293T packaging cells for lentivirus production, as well as the A549 human lung adenocarcinoma cell line were cultured in DMEM (Lonza, Basel, Switherland). Cell lines were tested routinely for mycoplasma contamination using the VenorRGeM-mycoplasma detection kit (Minerva Biolabs, Berlin, Germany). All reagents were purchased at Sigma-Aldrich (Vienna, Austria) unless indicated otherwise.

### Retroviral and lentiviral expression vectors

The retroviral vector pLIB-FOXO3(A3)-ER-iresNeo has been described [20]. The lentiviral plasmids coding for human FOXO3-specific shRNA (pLKO-shFOXO3-91616) and the control vector pLKO.1 were purchased at Sigma-Aldrich (Vienna, Austria).

### Production of lentiviruses for infection

Lentiviruses were produced as described previously [38]. NB8 cells were infected with the empty pLKO.1 as well as the pLKO.1-shFOXO3-91616 lentivirus-supernatant to generate NB8/shCtr and NB8/shFOXO3 cells, respectively.

### Generation and purification of recombinant FOXO-DBD proteins

DNA encoding human FOXO1-DBD (residues 159–272), FOXO3-DBD (residues 156–269), and FOXO6-DBD (residues 87–200) were codon-usage optimized, synthesized either by Genescript (George Town, Cayman Islands) or BioCat (Heidelberg, Germany), and ligated into the pGEX-6P-1 plasmid (GE Healthcare, Chicago, USA) using the BamHI and XhoI sites. The FOXO4-DBD (residues 82–207) expression plasmid was kindly provided by T. Obsil [78]. FOXO1-DBD, FOXO3-DBD, and FOXO6-DBD were expressed as N-terminal GST-tagged fusion protein; FOXO4-DBD was N-terminal tagged with histidins only in *E. coli* BL21(DE3)pLysS. Bacteria were expanded in LB-medium at 37 °C until the optical density reached OD600 0.6–0.8. Protein production of FOXO1-DBD, FOXO4-DBD, and FOXO6-DBD was induced with 0.5 M IPTG at 22 °C O/N, respectively; FOXO3-DBD production was induced with 1 M IPTG at 20 °C for eight hours. For protein isolation the bacterial pellet was resuspended in lysis buffer (1× PBS, 1 M NaCl, 4 mM β-mercaptoethanol (βME), 2 mM imidazole, 0.1 mg/ml lysozyme, 1 mM PMSF, and 1 tablet complete Mini (Roche, Mannheim, Germany)). After sonication the suspension was filtered through a 0.45 µm filter and applied to protein purification by HIS- and GST-tag chromatography. In a first step affinity chromatography was performed with the HisTrap™ High Performance column (GE Healthcare, Vienna, Austria), according to the manufacturer’s instructions. After equilibration with buffer containing 0.5 mM NaCl, 2 mM βME, and 1 mM imidazole, the protein lysate was injected onto the column. The column was washed (0.05 mM NaCl, 0.2 mM βME, 60 mM imidazole; pH 8.0) and the protein was eluted by applying elution buffer containing 0.5 mM NaCl, 2 mM βME, and 600 mM imidazole (pH 8.0). In a second step the protein was further purified with the GSTrap™ High Performance column (GE Healthcare, Vienna, Austria), according to the manufacturer’s instructions. The column was equilibrated with wash buffer containing 50 mM Tris-HCl (pH 7.5), 300 mM NaCl, 10% glycerol, 1 mM EDTA, and 1 mM DTT. The protein was injected onto the column and after applying wash buffer, the protein was eluted with buffer containing 50 mM Tris-HCl, 150 mM NaCl, 20 mM glutathione (GSH), 10% glycerol, 1 mM EDTA, and 1 mM DTT adjusted to pH 7.5. After buffer exchange using PD10 desalting columns (GE Healthcare, Chicago, USA), concentrations of distinct proteins were monitored at UV 280 with the corresponding molecular extinctions coefficient with a NanoDrop ND-1000 UV-Vis spectrophotometer (PeqLab Biotechnologie, Erlangen, Germany). The purified proteins were stored at −70 °C in buffer containing 20 mM Tris-HCl (pH 7.5), 100 mM NaCl, 1 mM EDTA, 1 mM DTT, and 10% glycerol.

### Fluorescence polarization assay (FPA)

The Prestwick Chemical Library^®^ (Illkirch, France) containing 1120 FDA-approved drugs was screened for compounds with FOXO3-DBD-interacting capacity, using oligonucleotides specific for the FOXO3 insulin-responsive sequence (IRE) (IRE-forward: CTATCAAAACAACGC, IRE-reverse: GCGTTGTTTTGATAG; Sigma-Aldrich, Vienna, Austria) [28]. The FPA was performed in black 96-well plates with flat bottom (Thermo Scientific, Huntsville, USA) pre-coated with 100 µl assay buffer (200 mM Tris pH 7.5, 100 mM NaCl, 1 mM EDTA) containing 100 µg/ml bovine serum albumin (BSA). For the screen, 1 µl of each compound (10 mg/ml) was incubated for 30 min at room temperature with 100 µl FOXO3/IRE-FAM mix containing recombinant FOXO3-DBD protein (100 nM) and the IRE-FAM oligonucleotide (5 nM) in assay buffer.

Negative (CTR; FOXO3-DBD protein and IRE-FAM oligonucleotide) and positive (IRE; FOXO3-DBD protein, IRE-FAM oligonucleotide in combination with 0.5 µM unlabeled IRE oligonucleotide) controls were analyzed on each plate. mP values were measured at an excitation wavelength of 485 nm and an emission wavelength of 530 nm in a chameleon plate reader (Hidex, Turku, Finland). Specificity of compound binding was verified by measuring the interaction between recombinant 14-3-3 sigma protein and R18 peptide [30, 31]. Comparison of the binding affinity of CBX to distinct FOXO-DBDs was performed in uncoated black 96-well plates. Therefore 125 nM FOXO1-DBD, FOXO3-DBD, FOXO4-DBD, or FOXO6-DBD were incubated with 25 nM IRE-FAM alone or in combination with 10, 20, or 80 µM CBX for 30 min at 15 °C.

### Determination of the equilibrium dissociation constant (K_d_), IC50, and binding affinity (K_i_) value

The equilibrium dissociation constant (K_d_) was determined by FPA. Increasing concentrations of FOXO3-DBD protein (1–350 nM) and 5 nM IRE-FAM oligonucleotide were used. The K_d_ was calculated by nonlinear curve fitting as the concentration of FOXO3-DBD protein at which 50% of the ligand is bound. The mP-values were plotted as function of the FOXO3-DBD protein concentration (log). To determine the IC50-value, 1–200 µM CBX were incubated with 25 nM FOXO3-DBD and 5 nM IRE oligonucleotide for 30 min at room temperature and FPA was performed. FPA readings were plotted as a function of the substance concentration (log) and the IC50-value was calculated by nonlinear least-square analysis. The K_i_ value of CBX was calculated by the equation of Nikolovska–Coleska [34]. All data analyses were done with GraphPad Prism 7.0 software (GraphPad Software, San Diego, USA).

### Fluorescence-based electrophoretic mobility shift assay (FAM-EMSA)

Fluorescence-labeled, double-stranded 100 nM FoxP3 or FoxP3-mut oligonucleotides (FoxP3 forward: 5′-AGCAAAGTTGTTTTTGATAATG-3′, reverse: 5′-CATTATCAAAAACAACTTTGCT-3′; FoxP3-mut forward: 5′-AGCAAAGTTGGGGTTGATAATG-3′, reverse: 3′-CATTATCAACCCCAACTTTGCT-5′; Microsynth, Balgach, Switzerland) [35, 36] were incubated with 1 µM recombinant FOXO3-DBD protein and CBX in assay buffer (20 mM Tris pH 7.5, 100 mM NaCl, 1 mM EDTA, 5% glycerol) for 30 min at room temperature. For the cell-based FAM-EMSA, 30 µg whole-cell extracts of SH-EP/FOXO3 cells treated with 50 nM 4OHT in combination with CBX were incubated with fluorescence-labeled, double-stranded FoxP3 oligonucleotides (100 nM) in assay buffer for 30 min at room temperature.

The samples were resolved on a 5% polyacrylamide gel in 0.5× TBE running buffer (45 mM Tris-HCL (pH 8.3), 45 mM boric acid, 1.3 mM EDTA). The fluorescence signal was analyzed with the Typhoon 9410 scanner (GE Healthcare, Vienna, Austria).

### Docking studies

Docking was performed using GOLD version 5.2 [79] and chain A of the PDB entry 2UZK [33]. The protein was protonated and the area of 20 Å around His212 was defined as binding site. The output was restricted to a maximum of 10 poses, which were ranked according to the GoldScore. Docking results were analyzed and visualized in LigandScout 3.12 [80].

### Chromatin immunoprecipitation assay (ChIP)

ChIP was performed using the Millipore Magna ChIP Kit (Millipore, Darmstadt, Germany) as described previously [38]. Protein-G-magnetic-beads (20 μl) were coupled to 2.5 μl of FOXO3 antibody (Santa Cruz Biotechnology, Dallas, USA) and incubated with nuclear lysates of shredded DNA from 2 × 10^7^ SH-EP/FOXO3 cells treated with 100 nM 4OHT alone or in combination with CBX for three hours. After precipitation, protein was digested by proteinase-K. Quantitative real-time RT-PCR analyses were performed to measure binding of FOXO3 to the DEPP-promoter (DEPP-forward: CTGCTCCTAGGAGAGACACACCCTG, DEPP-reverse: CTGCTACGTTTGCTGTGCTTAGTGC).

### Luciferase activity assay

To determine CBX-mediated inhibition of FOXO3 binding to the DEPP- and BIM-promoter, a luciferase reporter plasmid containing the DEPP-promoter [38] or the BIM-promoter [39] was transiently transfected into SH-EP/FOXO3 cells using the JetPrime^®^ Reagent (Polyplus, Berkeley, USA) according to the manufacturer’s instructions. Subsequently the cells were cultured in the presence of 4OHT alone, or in combination with increasing concentrations of CBX for eight hours. Luciferase activity was measured with the Luciferase Assay System kit (Promega, Madison, USA) according to the manufacturer’s instructions. The luminescence signal was measured with the chameleon plate reader (Hidex, Turku, Finland).

### Quantitative RT-PCR analysis

To quantify DEPP and BIM mRNA levels, we designed “real-time” RT-PCR assays, using GAPDH as reference gene. Total RNA was prepared from 5 × 10^6^ cells using TRIzol^TM^ Reagent (Invitrogen, Carlsbad, USA) according to the manufacturer’s instructions. cDNA was synthesized from 1 μg of total RNA using the Revert H Minus First Strand cDNA Synthesis Kit (Thermo Scientific, Huntsville, USA). Quantitative RT-PCR was performed as described previously [41] using DEPP-(forward ACTGTCCCTGCT CATCCATTCTC and reverse AGTCATCCAGGCTAGGAGAGGG), BIM-(forward AGCACCCATGAGTTGTGACAAATC and reverse CGTTAAACTCGTCTCCAATACGC), and GAPDH-specific oligonucleotides (forward TGTTCGTCATGGGTGTGAACC and reverse GCAGTGATGGCATGGACTGTG). After normalization on GAPDH expression, regulation was calculated between treated and untreated cells.

### Immunoblotting

Preparation of protein extracts was performed as described previously [40]. Immunoblots were incubated with antibodies specific to DEPP (25833-1-AP; Proteintech, Rosemont, USA); SESN3 (ab97792), FOXO6 (ab48730; Abcam, Cambridge, UK); FOXO1 (#2880), FOXO3 (#2497), FOXO4 (#9472), BIM (#2819), NOXA (#14766), P27KIP1 (#3686), FOXP1 (#4402), Lamin (#2032), Tubulin (#2144), and GAPDH (#2118; Cell Signaling, Danvers, USA). After incubation with anti-rabbit horseradish-peroxidase-conjugated secondary antibody (GE Healthcare, Vienna, Austria), the blots were developed by enhanced chemiluminescence (GE Healthcare, Vienna, Austria), and analyzed with an AutoChemi detection system (BioRad Laboratories, Munich, Germany). To generate positive-controls for antibody validation, plasmids for the respective overexpression of FOXO1 (Flag-FOXO1; provided by D. Accili; Addgene plasmid #12148), FOXO3 (Flag-FOXO3; provided by J. Massague; Addgene plasmid #14937), and FOXO4 (Flag-FOXO4; provided by D. Accili; Addgene plasmid #17549) were transiently transfected into SH-EP cells using the JetPrime^®^ Reagent (Polyplus, Berkeley, USA).

### Determination of apoptosis by flow cytometry

Apoptosis was measured by staining the cells with propidium-iodide (PI)/Triton X-100 and forward/sideward scatter analysis using a CytomicsFC-500 Beckman Coulter. A total of 2 × 10^5^ cells were harvested and incubated in 500 μl hypotonic PI solution containing 0.1% Triton X-100 for up to four hours at 4 °C. Stained nuclei in the sub-G1 marker window were considered to represent apoptotic cells.

### Colony formation assay

1 × 10^6^ NB4/FOXO3 or NB8/FOXO3 cells were seeded and pre-treated with 50 nM 4OHT and 80 µM CBX for 72 hours prior to the combined treatment with etoposide or doxorubicin for another 72 hours. When untreated controls reached 100% confluence, colonies were fixed with ice-cold methanol, stained with 0.2% crystal violet in 50% methanol, and quantified by photometry at 600 nm after discoloration with 0.5% SDS in 50% ethanol.

### Microtissue culture

For the production of 3D spheroids, the GravityPLUS™ microtissue culture system (InSphero AG, Zürich, Switzerland) was used. A total of 2 × 10^4^ cells were seeded in 40 μl drops into the hanging-drop plates and grown for 96 hours to form spheroids. Size was monitored regularly by live-cell microscopy. Subsequently, the spheroids were treated with etoposide alone or in combination with CBX for another 96 hours. Viable cells were stained with 2 µM calcein-AM for two hours at 37 °C. Representative live-cell images of the spheroids were taken with the DMi8 inverted microscope (Leica, Germany) and processed with the LAS X1.1.0 software (Leica, Germany). Quantification of living cells was done using the CellTiter-Glo 3D^®^ cell viability assay according to the manufacturer’s instructions (Promega, Madison, USA). Briefly, single spheroids were collected in 50 µl RPMI1640 media and incubated with 50 µl CellTiter-Glo 3D^®^ cell viability reagent for 30 min at room temperature. The luminescence signal was measured with the chameleon plate reader (Hidex, Turku, Finland).

### Caspase 3/7 assay

Caspase 3/7 activity was determined using the Caspase-Glo 3/7 assay kit according to the manufacturer’s instructions (Promega, Madison, USA). Briefly, cells were cultured at 80% confluence. After treatment, 50 µl of the supernatant was transferred to white 96-well plates and incubated with 50 µl Caspase-Glo^®^ 3/7 substrate for one hour at room temperature. Luminescence of each sample was analyzed with a Hidex Sense microplate reader (Hidex, Turku, Finland). Caspase 3/7 activity was calculated between treated and untreated cells.

### Statistics

Statistical significance of differences between the means were assessed using Student’s unpaired *t* test.

## Supplementary information

Supplemental figure legends

Supplemental Figure S1

Supplemental Figure S2

Supplemental Figure S3

Supplemental Figure S4

Supplemental Figure S5

## References

[1] Maris JM, Hogarty MD, Bagatell R, Cohn SL (2007). Neuroblastoma. Lancet.

[2] Coomans de Brachene A, Demoulin JB (2016). FOXO transcription factors in cancer development and therapy. Cell Mol Life Sci.

[3] Ausserlechner MJ, Hagenbuchner J, Fuchs S, Geiger K, Obexer P. FOXO transcription factors as potential therapeutic targets in neuroblastoma. In Shimada H, editor Neuroblastoma—present and future. Rijeka, Croatia: INTECH; 2012. pp. 325–48.

[4] Hui RC, Gomes AR, Constantinidou D, Costa JR, Karadedou CT, Fernandez de Mattos S (2008). The forkhead transcription factor FOXO3a increases phosphoinositide-3 kinase/Akt activity in drug-resistant leukemic cells through induction of PIK3CA expression. Mol Cell Biol.

[5] Hui RC, Francis RE, Guest SK, Costa JR, Gomes AR, Myatt SS (2008). Doxorubicin activates FOXO3a to induce the expression of multidrug resistance gene ABCB1 (MDR1) in K562 leukemic cells. Mol Cancer Ther.

[6] Tenbaum SP, Ordonez-Moran P, Puig I, Chicote I, Arques O, Landolfi S (2012). Beta-catenin confers resistance to PI3K and AKT inhibitors and subverts FOXO3a to promote metastasis in colon cancer. Nat Med.

[7] Storz P, Doppler H, Copland JA, Simpson KJ, Toker A (2009). FOXO3a promotes tumor cell invasion through the induction of matrix metalloproteinases. Mol Cell Biol.

[8] Chen J, Gomes AR, Monteiro LJ, Wong SY, Wu LH, Ng TT (2010). Constitutively nuclear FOXO3a localization predicts poor survival and promotes Akt phosphorylation in breast cancer. PLoS ONE.

[9] Pellicano F, Scott MT, Helgason GV, Hopcroft LE, Allan EK, Aspinall-O’Dea M (2014). The antiproliferative activity of kinase inhibitors in chronic myeloid leukemia cells is mediated by FOXO transcription factors. Stem Cells.

[10] Naka K, Hoshii T, Muraguchi T, Tadokoro Y, Ooshio T, Kondo Y (2010). TGF-beta-FOXO signalling maintains leukaemia-initiating cells in chronic myeloid leukaemia. Nature.

[11] Santamaria CM, Chillon MC, Garcia-Sanz R, Perez C, Caballero MD, Ramos F (2009). High FOXO3a expression is associated with a poorer prognosis in AML with normal cytogenetics. Leuk Res.

[12] Sykes SM, Lane SW, Bullinger L, Kalaitzidis D, Yusuf R, Saez B (2011). AKT/FOXO signaling enforces reversible differentiation blockade in myeloid leukemias. Cell.

[13] Hurtz C, Hatzi K, Cerchietti L, Braig M, Park E, Kim YM (2011). BCL6-mediated repression of p53 is critical for leukemia stem cell survival in chronic myeloid leukemia. J Exp Med.

[14] Kumazoe M, Takai M, Hiroi S, Takeuchi C, Kadomatsu M, Nojiri T (2017). The FOXO3/PGC-1beta signaling axis is essential for cancer stem cell properties of pancreatic ductal adenocarcinoma. J Biol Chem.

[15] Kumazoe M, Takai M, Bae J, Hiroi S, Huang Y, Takamatsu K (2017). FOXO3 is essential for CD44 expression in pancreatic cancer cells. Oncogene.

[16] Xu K, Zhang Z, Pei H, Wang H, Li L, Xia Q (2017). FoxO3a induces temozolomide resistance in glioblastoma cells via the regulation of beta-catenin nuclear accumulation. Oncol Rep.

[17] Qian Z, Ren L, Wu D, Yang X, Zhou Z, Nie Q (2017). Overexpression of FoxO3a is associated with glioblastoma progression and predicts poor patient prognosis. Int J Cancer.

[18] Hagenbuchner J, Rupp M, Salvador C, Meister B, Kiechl-Kohlendorfer U, Müller T (2016). Nuclear FOXO3 predicts adverse clinical outcome and promotes tumor angiogenesis in neuroblastoma. Oncotarget.

[19] Rupp M, Hagenbuchner J, Rass B, Fiegl H, Kiechl-Kohlendorfer U, Obexer P (2017). FOXO3-mediated chemo-protection in high-stage neuroblastoma depends on wild-type TP53 and SESN3. Oncogene.

[20] Obexer P, Geiger K, Ambros PF, Meister B, Ausserlechner MJ (2007). FKHRL1-mediated expression of Noxa and Bim induces apoptosis via the mitochondria in neuroblastoma cells. Cell Death Differ.

[21] Opel D, Poremba C, Simon T, Debatin KM, Fulda S (2007). Activation of Akt predicts poor outcome in neuroblastoma. Cancer Res.

[22] Naka K, Hoshii T, Hirao A (2010). Novel therapeutic approach to eradicate tyrosine kinase inhibitor resistant chronic myeloid leukemia stem cells. Cancer Sci.

[23] Komatsu N, Watanabe T, Uchida M, Mori M, Kirito K, Kikuchi S (2003). A member of Forkhead transcription factor FKHRL1 is a downstream effector of STI571-induced cell cycle arrest in BCR-ABL-expressing cells. J Biol Chem.

[24] Osuka S, Sampetrean O, Shimizu T, Saga I, Onishi N, Sugihara E (2013). IGF1 receptor signaling regulates adaptive radioprotection in glioma stem cells. Stem Cells.

[25] Yu Y, Guo M, Wei Y, Yu S, Li H, Wang Y (2016). FoxO3a confers cetuximab resistance in RAS wild-type metastatic colorectal cancer through c-Myc. Oncotarget.

[26] Wilson MS, Brosens JJ, Schwenen HD, Lam EW (2011). FOXO and FOXM1 in cancer: the FOXO-FOXM1 axis shapes the outcome of cancer chemotherapy. Curr Drug Targets.

[27] Zhou Y, Chen Y, Ding W, Hua Z, Wang L, Zhu Y (2018). LncRNA UCA1 impacts cell proliferation, invasion, and migration of pancreatic cancer through regulating miR-96/FOXO3. IUBMB Life.

[28] Obsil T, Obsilova V (2011). Structural basis for DNA recognition by FOXO proteins. Biochim Biophys Acta.

[29] Sanna PP, Kawamura T, Chen J, Koob GF, Roberts AJ, Vendruscolo LF (2016). 11beta-hydroxysteroid dehydrogenase inhibition as a new potential therapeutic target for alcohol abuse. Transl Psychiatry.

[30] Du Y, Khuri FR, Fu H (2008). A homogenous luminescent proximity assay for 14-3-3 interactions with both phosphorylated and nonphosphorylated client peptides. Curr Chem Genomics.

[31] Du Y, Masters SC, Khuri FR, Fu H (2006). Monitoring 14-3-3 protein interactions with a homogeneous fluorescence polarization assay. J Biomol Screen.

[32] Obsil T, Obsilova V (2008). Structure/function relationships underlying regulation of FOXO transcription factors. Oncogene.

[33] Tsai KL, Sun YJ, Huang CY, Yang JY, Hung MC, Hsiao CD (2007). Crystal structure of the human FOXO3a-DBD/DNA complex suggests the effects of post-translational modification. Nucleic Acids Res.

[34] Nikolovska-Coleska Z, Wang R, Fang X, Pan H, Tomita Y, Li P (2004). Development and optimization of a binding assay for the XIAP BIR3 domain using fluorescence polarization. Anal Biochem.

[35] Harada Y, Harada Y, Elly C, Ying G, Paik JH, DePinho RA (2010). Transcription factors Foxo3a and Foxo1 couple the E3 ligase Cbl-b to the induction of Foxp3 expression in induced regulatory T cells. J Exp Med.

[36] Ouyang W, Beckett O, Ma Q, Paik JH, DePinho RA, Li MO (2010). Foxo proteins cooperatively control the differentiation of Foxp3+ regulatory T cells. Nat Immunol.

[37] Hou J, Wang Z, Yue Y, Li Q, Shao S (2015). Spectroscopic analysis on structure-affinity relationship in the interactions of different oleanane-type triterpenoids with bovine serum albumin. Luminescence.

[38] Salcher S, Hagenbuchner J, Geiger K, Seiter MA, Rainer J, Kofler R (2014). C10ORF10/DEPP, a transcriptional target of FOXO3, regulates ROS-sensitivity in human neuroblastoma. Mol Cancer.

[39] Bouillet P, Zhang LC, Huang DC, Webb GC, Bottema CD, Shore P (2001). Gene structure alternative splicing, and chromosomal localization of pro-apoptotic Bcl-2 relative Bim. Mamm Genome.

[40] Salcher S, Hermann M, Kiechl-Kohlendorfer U, Ausserlechner MJ, Obexer P (2017). C10ORF10/DEPP-mediated ROS accumulation is a critical modulator of FOXO3-induced autophagy. Mol Cancer.

[41] Hagenbuchner J, Kuznetsov A, Hermann M, Hausott B, Obexer P, Ausserlechner MJ (2012). FOXO3-induced reactive oxygen species are regulated by BCL2L11 (Bim) and SESN3. J Cell Sci.

[42] Medema RH, Kops GJ, Bos JL, Burgering BM (2000). AFX-like Forkhead transcription factors mediate cell-cycle regulation by Ras and PKB through p27kip1. Nature.

[43] van Boxtel R, Gomez-Puerto C, Mokry M, Eijkelenboom A, van der Vos KE, Nieuwenhuis EE (2013). FOXP1 acts through a negative feedback loop to suppress FOXO-induced apoptosis. Cell Death Differ.

[44] Bertaccini G, Coruzzi G (1985). Pharmacology of the treatment of peptic ulcer disease. Dig Dis Sci.

[45] O’Carroll SJ, Becker DL, Davidson JO, Gunn AJ, Nicholson LF, Green CR (2013). The use of connexin-based therapeutic approaches to target inflammatory diseases. Methods Mol Biol.

[46] Moosavi MA, Moasses Ghafary S, Asvadi-Kermani I, Hamzeiy H, Rahmati M, Ahmadi AH (2011). Carbenoxolone induces apoptosis and inhibits survivin and survivin-DeltaEx3 genes expression in human leukemia K562 cells. Daru.

[47] Yulyana Y, Endaya BB, Ng WH, Guo CM, Hui KM, Lam PY (2013). Carbenoxolone enhances TRAIL-induced apoptosis through the upregulation of death receptor 5 and inhibition of gap junction intercellular communication in human glioma. Stem Cells Dev.

[48] Jiang JX, Penuela S (2016). Connexin and pannexin channels in cancer. BMC Cell Biol.

[49] Maiese K (2016). Forkhead transcription factors: new considerations for alzheimer’s disease and dementia. J Transl Sci.

[50] Shi C, Viccaro K, Lee HG, Shah K (2016). Cdk5-Foxo3 axis: initially neuroprotective, eventually neurodegenerative in Alzheimer’s disease models. J Cell Sci.

[51] Zeldich E, Chen CD, Colvin TA, Bove-Fenderson EA, Liang J, Tucker Zhou TB (2014). The neuroprotective effect of Klotho is mediated via regulation of members of the redox system. J Biol Chem.

[52] Wong HK, Veremeyko T, Patel N, Lemere CA, Walsh DM, Esau C (2013). De-repression of FOXO3a death axis by microRNA-132 and -212 causes neuronal apoptosis in Alzheimer’s disease. Hum Mol Genet.

[53] Qin W, Zhao W, Ho L, Wang J, Walsh K, Gandy S (2008). Regulation of forkhead transcription factor FoxO3a contributes to calorie restriction-induced prevention of Alzheimer’s disease-type amyloid neuropathology and spatial memory deterioration. Ann NY Acad Sci.

[54] Saha P, Biswas SC (2015). Amyloid-beta induced astrocytosis and astrocyte death: implication of FoxO3a-Bim-caspase3 death signaling. Mol Cell Neurosci.

[55] Shi C, Zhu J, Leng S, Long D, Luo X (2016). Mitochondrial FOXO3a is involved in amyloid beta peptide-induced mitochondrial dysfunction. J Bioenerg Biomembr.

[56] Sun L, Zhao M, Liu M, Su P, Zhang J, Li Y (2018). Suppression of FoxO3a attenuates neurobehavioral deficits after traumatic brain injury through inhibiting neuronal autophagy. Behav Brain Res.

[57] Li D, Li X, Wu J, Li J, Zhang L, Xiong T (2015). Involvement of the JNK/FOXO3a/Bim pathway in neuronal apoptosis after hypoxic-ischemic brain damage in neonatal rats. PLoS ONE.

[58] Li D, Qu Y, Mao M, Zhang X, Li J, Ferriero D (2009). Involvement of the PTEN-AKT-FOXO3a pathway in neuronal apoptosis in developing rat brain after hypoxia-ischemia. J Cereb Blood Flow Metab.

[59] Yoo KY, Kwon SH, Lee CH, Yan B, Park JH, Ahn JH (2012). FoxO3a changes in pyramidal neurons and expresses in non-pyramidal neurons and astrocytes in the gerbil hippocampal CA1 region after transient cerebral ischemia. Neurochem Res.

[60] Sandeep TC, Yau JL, MacLullich AM, Noble J, Deary IJ, Walker BR (2004). 11Beta-hydroxysteroid dehydrogenase inhibition improves cognitive function in healthy elderly men and type 2 diabetics. Proc Natl Acad Sci USA.

[61] Zhou LQ, Liu CL, Wang Z, Shen HT, Wen ZJ, Chen DD (2018). Pannexin-1 is involved in neuronal apoptosis and degeneration in experimental intracerebral hemorrhage in rats. Mol Med Rep.

[62] Takeuchi H, Mizoguchi H, Doi Y, Jin S, Noda M, Liang J (2011). Blockade of gap junction hemichannel suppresses disease progression in mouse models of amyotrophic lateral sclerosis and Alzheimer’s disease. PLoS ONE.

[63] Pahrudin Arrozi A, Shukri SNS, Wan Ngah WZ, Mohd Yusof YA, Ahmad Damanhuri MH, Makpol S (2017). Evaluation of the expression of amyloid precursor protein and the ratio of secreted amyloid beta 42 to amyloid beta 40 in SH-SY5Y cells stably transfected with wild-type, single-mutant and double-mutant forms of the APP gene for the study of alzheimer’s disease pathology. Appl Biochem Biotechnol.

[64] Bingel C, Koeneke E, Ridinger J, Bittmann A, Sill M, Peterziel H (2017). Three-dimensional tumor cell growth stimulates autophagic flux and recapitulates chemotherapy resistance. Cell death Dis.

[65] Baron JH, Gribble JN, Rhodes C, Wright PA (1978). Serum carbenoxolone in patients with gastric and duodenal ulcer: absorption, efficacy and side-effects. Gut.

[66] Nagashima T, Shigematsu N, Maruki R, Urano Y, Tanaka H, Shimaya A (2010). Discovery of novel forkhead box O1 inhibitors for treating type 2 diabetes: improvement of fasting glycemia in diabetic db/db mice. Mol Pharm.

[67] Savai R, Al-Tamari HM, Sedding D, Kojonazarov B, Muecke C, Teske R (2014). Pro-proliferative and inflammatory signaling converge on FoxO1 transcription factor in pulmonary hypertension. Nat Med.

[68] Zou P, Liu L, Zheng L, Liu L, Stoneman RE, Cho A (2014). Targeting FoxO1 with AS1842856 suppresses adipogenesis. Cell Cycle.

[69] Karki S, Farb MG, Ngo DT, Myers S, Puri V, Hamburg NM (2015). Forkhead box O-1 modulation improves endothelial insulin resistance in human obesity. Arterioscler Thromb Vasc Biol.

[70] Tan P, Guan H, Xie L, Mi B, Fang Z, Li J (2015). FOXO1 inhibits osteoclastogenesis partially by antagnozing MYC. Sci Rep.

[71] Matkar S, Sharma P, Gao S, Gurung B, Katona BW, Liao J (2015). An epigenetic pathway regulates sensitivity of breast cancer cells to HER2 inhibition via FOXO/c-Myc axis. Cancer Cell.

[72] Diep CH, Charles NJ, Gilks CB, Kalloger SE, Argenta PA, Lange CA (2013). Progesterone receptors induce FOXO1-dependent senescence in ovarian cancer cells. Cell Cycle.

[73] Pan CW, Jin X, Zhao Y, Pan Y, Yang J, Karnes RJ (2017). AKT-phosphorylated FOXO1 suppresses ERK activation and chemoresistance by disrupting IQGAP1-MAPK interaction. EMBO J.

[74] Baar MP, Brandt RMC, Putavet DA, Klein JDD, Derks KWJ, Bourgeois BRM (2017). Targeted apoptosis of senescent cells restores tissue homeostasis in response to chemotoxicity and aging. Cell.

[75] Narath R, Lorch T, Greulich-Bode KM, Boukamp P, Ambros PF (2005). Automatic telomere length measurements in interphase nuclei by IQ-FISH. Cytom Part A.

[76] Gross N, Favre S, Beck D, Meyer M (1992). Differentiation-related expression of adhesion molecules and receptors on human neuroblastoma tissues, cell lines and variants. Int J Cancer.

[77] Grignani F, Kinsella T, Mencarelli A, Valtieri M, Riganelli D, Grignani F (1998). High-efficiency gene transfer and selection of human hematopoietic progenitor cells with a hybrid EBV/retroviral vector expressing the green fluorescence protein. Cancer Res.

[78] Boura E, Silhan J, Herman P, Vecer J, Sulc M, Teisinger J (2007). Both the N-terminal loop and wing W2 of the forkhead domain of transcription factor Foxo4 are important for DNA binding. J Biol Chem.

[79] Jones G, Willett P, Glen RC, Leach AR, Taylor R (1997). Development and validation of a genetic algorithm for flexible docking. J Mol Biol.

[80] Wolber G, Langer T (2005). LigandScout: 3-D pharmacophores derived from protein-bound ligands and their use as virtual screening filters. J Chem Inf Model.

