# Supplementary material for: A drug library screen identifies Carbenoxolone as novel FOXO inhibitor that overcomes FOXO3-mediated chemoprotection in high-stage neuroblastoma
Source: Oncogene. 2019 Oct 7;39(5):1080–97. doi: 10.1038/s41388-019-1044-7 (PMC6989399; doi:10.1038/s41388-019-1044-7)
Supplement: Supplementary file 1 — Supplemental figure legends [file 41388_2019_1044_MOESM1_ESM.docx]

**Supplemental Figure S1.** 1 µg recombinant GST-His purified FOXO3-DBD protein was analyzed by Coomassie blue stained SDS-PAGE.

**Supplemental Figure S2.** SH-EP/FOXO3 cells were treated for 48 hours with 20 nM 4OHT alone or in combination with indicated concentrations of verteporfin, cefalonium, benserazide, berberine, lysergol, benzbromarone, oxytetracycline, or repaglinide. PI-FACS analyses were performed to detect apoptotic cells. Shown are mean values ± s.e.m. of three independent experiments.

**Supplemental Figure S3.** (**a**) 5 µg of GST-His-purified FOXO1-DBD, FOXO3-DBD, and FOXO6-DBD, as well as His-purified FOXO4-DBD proteins were analyzed by Coomassie blue stained SDS-PAGE. (**b**) Analyses of the dose-dependent interaction of CBX with the FOXO1-DBD, FOXO3-DBD, FOXO4-DBD, and FOXO6-DBD proteins (125 nM) by FPA using 25 nM IRE-FAM oligonucleotide in combination with 5 µM unlabeled IRE oligonucleotide or with indicated concentrations of CBX (µM). Shown are means ± s.e.m. of three independent experiments. Statistical analysis was done using the Student’s unpaired t-test; ***P < 0.01 compared to the negative control (CTR). (**c**) Immunoblot analyses of FOXO1, FOXO3, FOXO4, and FOXO6 expression in NB1 and NB8 cells. SH-EP cells transfected with plasmids for the respective overexpression of FOXO1 (Flag-FOXO1), FOXO3 (Flag-FOXO3), and FOXO4 (Flag-FOXO4) were used as positive-controls for antibody validation of FOXO1, FOXO3, and FOXO4, respectively. Human A549 cells were loaded as positive-control for the FOXO6 antibody. GAPDH served as loading control.

**Supplemental Figure S4.** (**a**) Chemical structure of enoxolone. (**b**) Analyses of the interaction of CBX and enoxolone with the FOXO3-DBD protein (20 nM) by FPA. 5 nM IRE-FAM oligonucleotide were incubated in combination with 0.5 µM unlabeled IRE oligonucleotide or with 80 µM CBX or 80 µM enoxolone. Shown are means ± s.e.m. of three independent experiments. Statistical analysis was done using the Student’s unpaired t-test; ***P < 0.01 compared to the negative control (CTR). (**c**) Highest-ranked poses and annotated GoldScores of CBX and enoxolone (gray sticks) docked into the FOXO3-DNA interaction site (blue surface). Interactions with protein residues are highlighted as red arrows (hydrogen bond acceptors), dark red asterisks (negatively charged moieties involved in ionic interactions), and yellow spheres or areas (hydrophobic interactions). (**d**) SH-EP/FOXO3 cells were treated with 20 nM 4OHT and with the indicated concentrations of enoxolone for 48 hours. PI-FACS analyses were performed to detect apoptotic cells. Shown are mean values ± s.e.m. of three independent experiments.

**Supplemental Figure S5.** The impact of CBX on FOXO3-mediated DEPP expression in etoposide-treated cells was analyzed by quantitative RT-PCR (**a**) and by immunoblot analyses (**b**). For quantitative RT-PCR analysis of DEPP expression, SH-EP cells were treated with 20 µg/ml etoposide and with 120 µM CBX for six hours. Shown are means ± s.e.m. of three independent experiments. Statistical analysis was done with the Student’s unpaired t-test, *P < 0.05 compared to the corresponding control. (**b**) For immunoblot analyses, SH-EP cells were cultivated in the presence of 10 µg/ml etoposide and 120 µM CBX for eight hours. GAPDH served as loading control.
