# Supplementary figures and images for: A drug library screen identifies Carbenoxolone as novel FOXO inhibitor that overcomes FOXO3-mediated chemoprotection in high-stage neuroblastoma
Source: Oncogene. 2019 Oct 7;39(5):1080–97. doi: 10.1038/s41388-019-1044-7 (PMC6989399; doi:10.1038/s41388-019-1044-7)

# Supplemental Figure S1

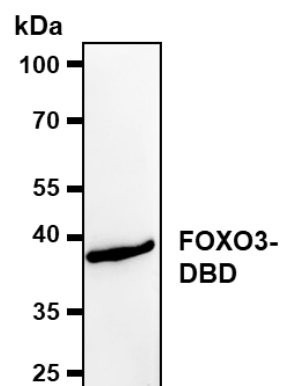

Supplement: Supplementary file 2 — Supplemental Figure S1 [file 41388_2019_1044_MOESM2_ESM.pdf]

Supplemental Figure S2

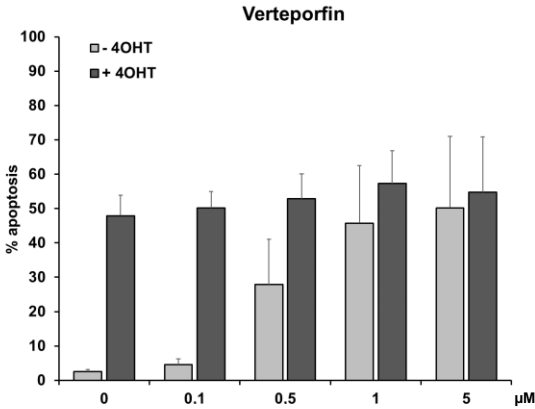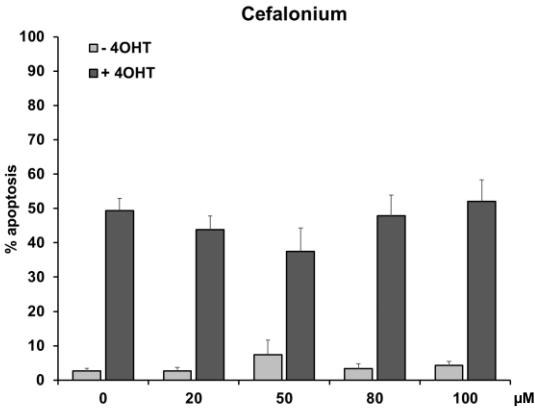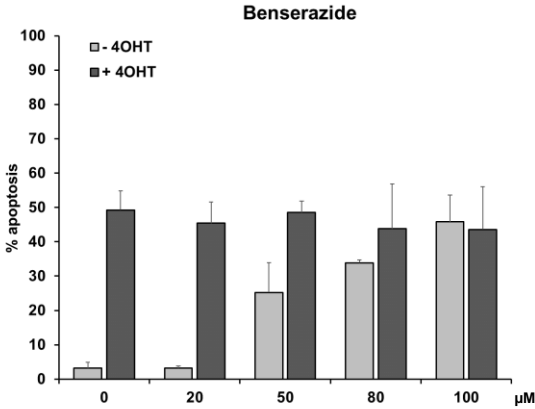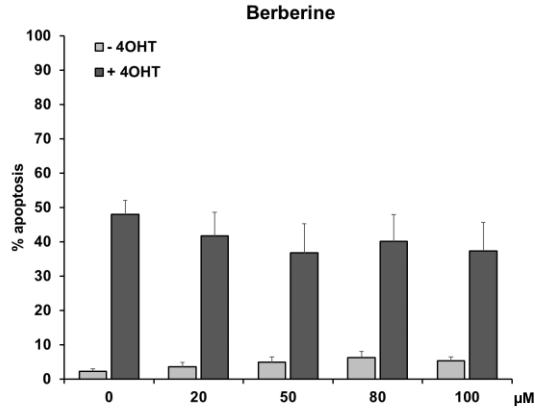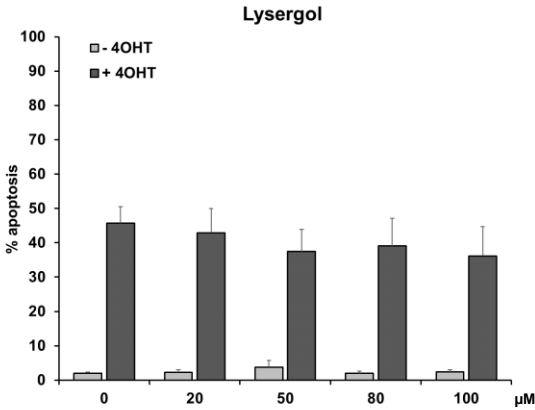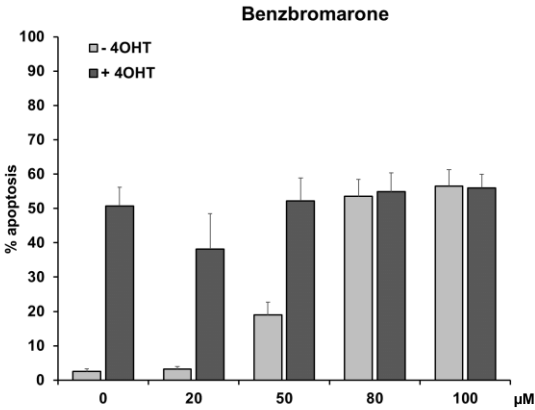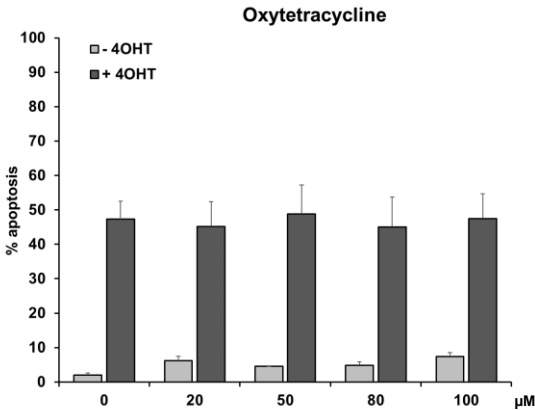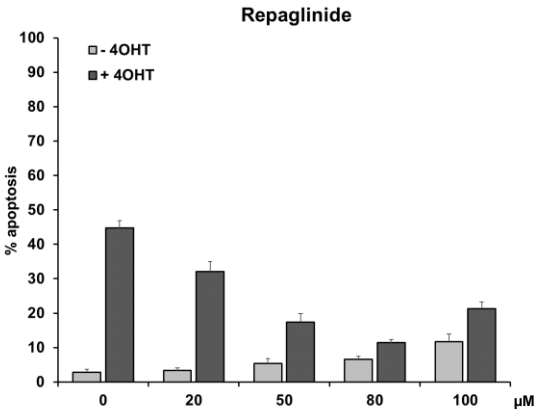

Supplement: Supplementary file 3 — Supplemental Figure S2 [file 41388_2019_1044_MOESM3_ESM.pdf]

Supplemental Figure S3

a

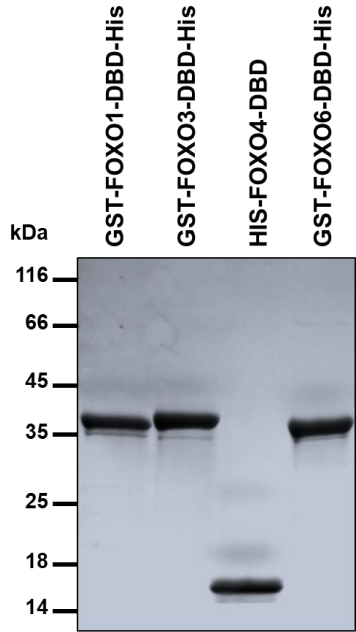

b

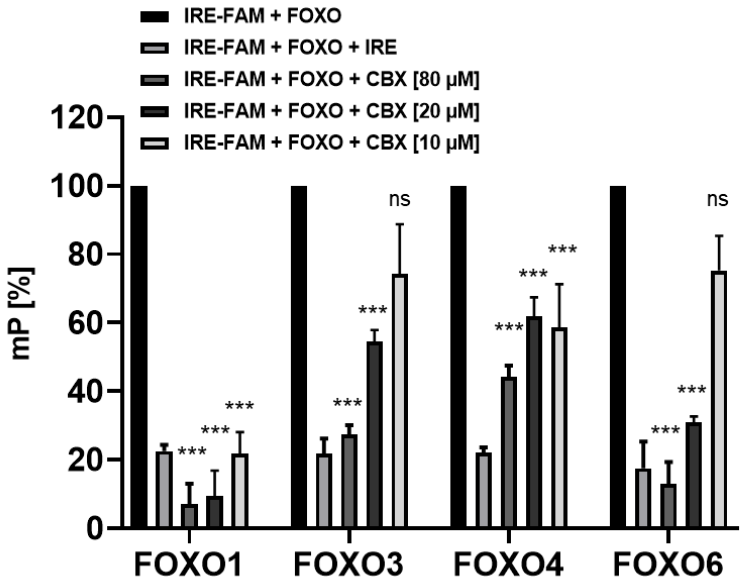

c

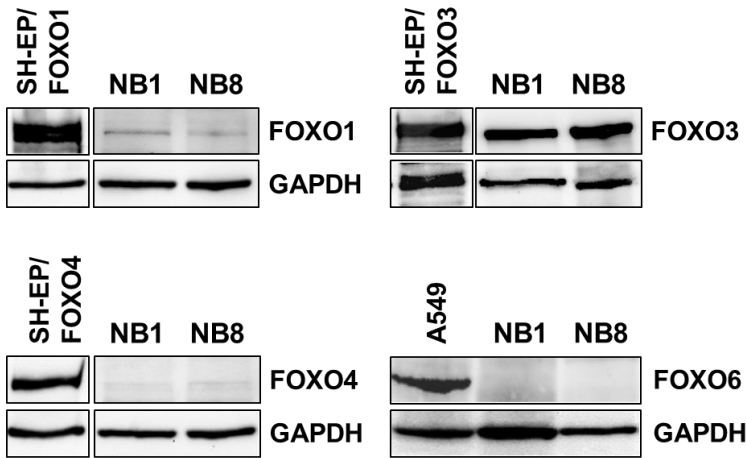

Supplement: Supplementary file 4 — Supplemental Figure S3 [file 41388_2019_1044_MOESM4_ESM.pdf]

Supplemental Figure S4

a

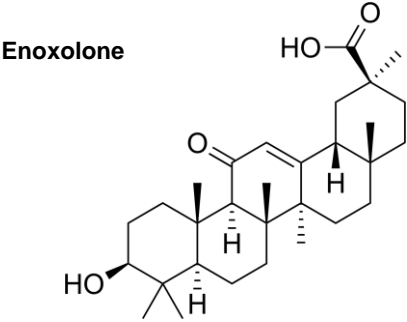

b

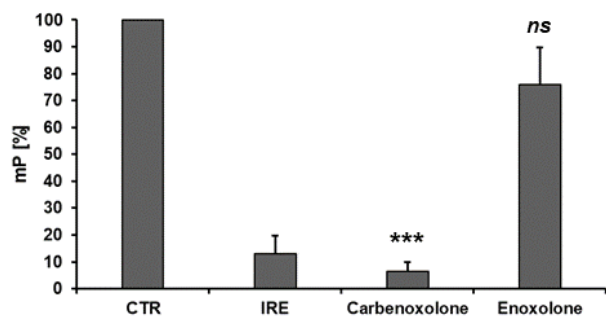

c

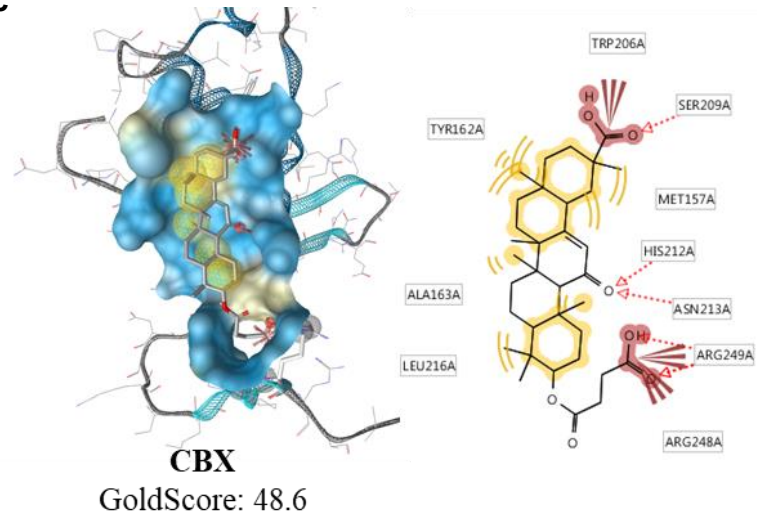

d

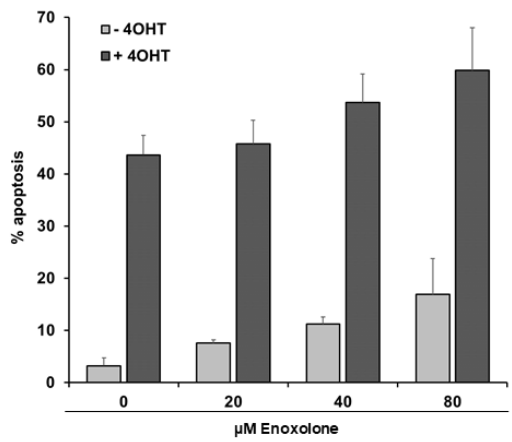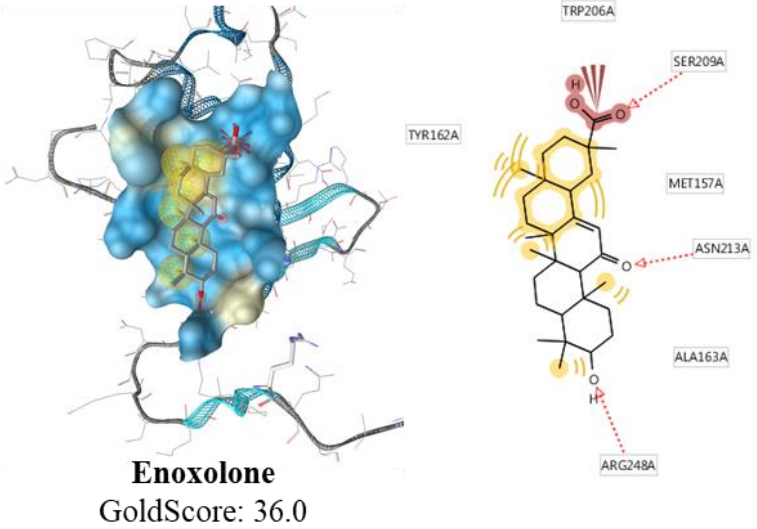

Supplement: Supplementary file 5 — Supplemental Figure S4 [file 41388_2019_1044_MOESM5_ESM.pdf]

Supplemental Figure S5

a

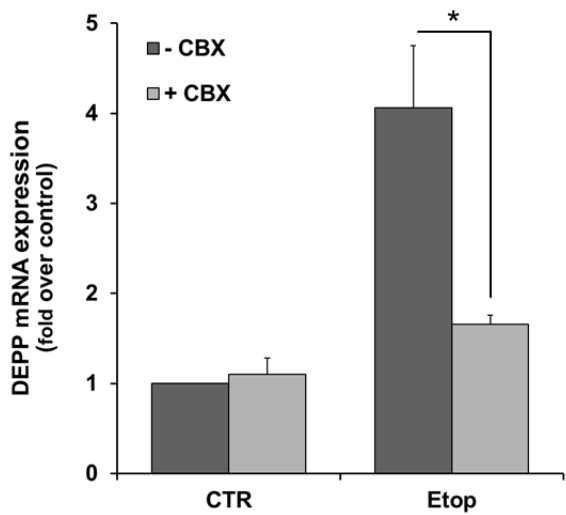

b

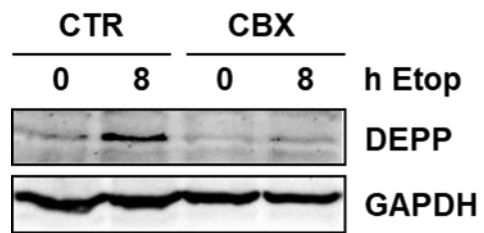

Supplement: Supplementary file 6 — Supplemental Figure S5 [file 41388_2019_1044_MOESM6_ESM.pdf]
